# Serotonin system is partially involved in immunomodulation of Nile tilapia (*Oreochromis niloticus*) immune cells

**DOI:** 10.3389/fimmu.2022.944388

**Published:** 2022-07-28

**Authors:** Qi Li, Baijian Jiang, Zhiqiang Zhang, Yongxiong Huang, Zhou Xu, Xinjin Chen, Xitan Hou, Jia Cai, Yu Huang, Jichang Jian

**Affiliations:** ^1^ College of Fishery, Guangdong Ocean University, Guangdong Provincial Key Laboratory of Aquatic Animal Disease Control and Healthy Culture, Zhanjiang, China; ^2^ Institute of Forensic Medicine and Laboratory Medicine, Jining Medical University, Jining, China; ^3^ Laboratory for Marine Biology and Biotechnology, Qingdao National Laboratory for Marine Science and Technology, Qingdao, China; ^4^ Guangdong Provincial Engineering Research Center for Aquatic Animal Health Assessment, Shenzhen, China

**Keywords:** Nile tilapia, serotonin, serotonin receptors, immunomodulation, *Streptococcus agalactiae*

## Abstract

Serotonin (5-hydroxytryptamine) is a well-known neurotransmitter affecting emotion, behavior, and cognition. Additionally, numerous immunomodulatory functions of serotonin have been discovered in mammals. However, the regulatory role of the serotonin system in fish immunity remains unclear. In this study, various serotonergic markers in Nile tilapia (*Oreochromis niloticus*) were identified and characterized. The involvement of the serotonin system during bacterial infection was investigated. Moreover, the expression characteristics and specific functions of serotonergic markers within Nile tilapia immune cells were also assessed. Overall, 22 evolutionarily conserved serotonergic marker genes in Nile tilapia were cloned and characterized. Transcriptional levels of these molecules were most abundant in the brain, and their transcripts were induced during *Streptococcus agalactiae* infection. Nevertheless, few serotonergic markers exist on Nile tilapia immune cells, and no distinct immunomodulation effect was observed during an immune response. The present study lays a theoretical foundation for further investigation of the immunological mechanisms in fish as well as the evolution of the serotonin system in animals.

## Introduction

Serotonin (5-hydroxytryptamine, 5-HT) is one of the most extensively studied neurotransmitters of the central nervous system (CNS) in vertebrates. It is known to regulate emotion, behavior, and cognition (1, 2). Besides, 5-HT plays an important role in many organs as a peripheral hormone (1, 3). The known evidences show that immune cells and cancer cells also synthesize, release, and respond to serotonin. The complex influence of serotonin on immune cells is becoming clearer (1, 2, 4).

The essential amino acid L-tryptophan provides the substrate for serotonin synthesis in the body, through a two-step process catalyzed by the enzymes tryptophan hydroxylase (TPH) and monoamine decarboxylase (5, 6). Additionally, the rate-limiting enzyme TPH exists in two isoforms (TPH1 and TPH2). In mammals, TPH1 is primarily expressed in enterochromaffin cells of the gastrointestinal mucosa, whereas TPH2 is located in central and enteric neurons. In the brain, serotonin mediates various neuro-psychotic processes. Meanwhile, peripheral serotonin, synthesized by enterochromaffin cells, is released into the blood. It is then mainly taken up by platelets *via* the sodium-dependent serotonin transporter (SERT, SLC6A4) and stored in dense granules at a high concentration (1, 7). Once platelets are activated at the site of injury, thrombus formation, or inflammation, the stored serotonin is rapidly released. Serotonin concentrations can rapidly increase from resting level (~10 nM) to >10 µM (1, 2, 8).

The function of serotonin is primarily mediated *via* binding to a serotonin receptor. There are seven known serotonin receptor (HTR) families, HTR1–HTR7 (including 14 distinct subtypes in humans) (1, 9–11). All HTRs are members of the G-protein coupled receptor superfamily, except for HTR3, which belongs to the Cys-loop ligand-gated ion channel family (12). The binding of serotonin with HTRs, could selectively activate four major signal pathways (PI3K/Akt, PKC/Ca^2+^, MAPK, and PKA-cAMP) (4), then serotonin is degraded by the monoamine oxidase (MAO), thereby producing 5-hydroxyindoleacetic acid to be excreted in the urine (2, 3).

As Mössner and Lesch discussed and verified, the two criteria for confirmed neural-immune interaction of serotonin *via* the autonomic nervous system are that HTRs must be present on immune cells and serotonin must have immunomodulatory effects (1, 3, 13). Recent studies indicate that nearly all immune cells express at least one serotonin component. Perhaps more interestingly, some immune cells, such as mast cells and T lymphocytes, have serotonin biosynthesis function (1, 2).

Additionally, the serotonin system and the evolutionary conservatism have been confirmed in the CNS from all vertebrate clades, including jawless, cartilaginous fish, and teleosts (14–16). Numerous core molecules involved in serotonin metabolism have been identified and characterized in fish, including TPH, SERT, MAO, and several HTRs (16–18). Additional serotonergic markers have been identified in teleosts such as HTR1a/b and SERTa/b in zebrafish (19), perhaps because of the two rounds of whole genome duplication in fish (20). Recently, the roles of serotonin in fish sleep (21), reproduction (14), neuron development, regeneration (22), social stress response (23), and bacterial infection (24) have been reported. Nevertheless, the exact immunomodulatory effects and their specific molecular mechanisms in fish immunity remain unclear.

Thus, in this research, a series of serotonergic markers including TPH, SERT, MAO, and HTRs have been identified and characterized in Nile tilapia (*Oreochromis niloticus*), an important cultured freshwater fish in South China (25). Moreover, their expression characteristics during bacterial infection caused by *Streptococcus agalatiae* were investigated, since the *S. agalatiae* infection usually leading to serious meningitis (26, 27). Nevertheless, neither an obvious presence of serotonergic markers on Nile tilapia immune cells nor a distinct immunomodulatory effect during the immune response was observed. These data emphasize the need to revisit the roles of serotonin in fish immunity and to expand our understanding of the evolutionary process of the serotonergic system among vertebrates.

## Materials and methods

### Fish rearing

All healthy Nile tilapia (100 ± 10 g) were obtained from a fish farm at Zhanjiang, Guangdong, China. The fish were acclimatized in a 1000 L tank with 20 fish per cubic meters of water for >60 days. Fish were fed with commercial feed daily (3% body weight). The water temperature was maintained at 28°C ± 0.5°C. The pH and dissolved oxygen remained within 7.3–7.8 and 5.0–6.0 mg/L, respectively. All fish for subsequent experiments were randomly selected, and all experiments were conducted following the Guangdong Province laboratory animal management regulations and granted by the Ethics Committee of the Guangdong Ocean University.

### RNA extraction and cDNA synthesis

Three healthy fish were anesthetized with 3-aminobenzoic acid ethyl ester methanesulfonate (MS-222, Sigma, Darmstadt, Germany). Next, eight tissues including brain, gills, head kidney, intestine, liver, muscle, skin, and spleen, were collected and the total RNA was immediately extracted. RNAiso Plus (TaKaRa, Dalian, China) was used to extract the total RNA following the manufacturer’s protocol. The total RNA quantity and purity were verified *via* electrophoresis using 1.2% agarose gels and measured using a NanoDrop 2000 (Thermo Fisher Scientific, Waltham, USA).

The RNA was reverse-transcribed *via* a PrimeScript™ RT reagent kit with gDNA Eraser (TaKaRa, Dalian, China) following the manufacturer’s instructions. The cDNA was then diluted with redistilled water at a ratio of 1:50 for subsequent experiments.

### Cloning and sequence analysis of serotonergic markers

All the primers (**Table S1**) used in this study were designed with the NCBI Primer designing tool (https://www.ncbi.nlm.nih.gov/tools/primer-blast/). The predicted gene sequences of serotonergic markers, including *TPH1*, *TPH2*, *SERT*, *MAO*, *HTR1A*, *HTR1B*, *HTR1D*, *HTR1E*, *HTR1F*, *HTR2A*, *HTR2B*, *HTR2C*, *HTR3A*, *HTR3B*, *HTR3C*, *HTR4*, *HTR5A*, *HTR6*, and *HTR7* were obtained from the Nile tilapia genome (GCF_001858045.2). The completed open reading frame (ORF) sequence was amplified *via* polymerase chain reaction (PCR) with cDNA of the brain (0 h) and specific primers. The PCRs were performed with *Premix Taq™* Hot Start Version (TaKaRa, Dalian, China) following the manufacturers’ instructions.

The multiple sequence alignments of putative proteins among various species were conducted using DNAMAN software (version 7.0). The maximum likelihood phylogenetic tree was constructed with MEGA software (version 6.0). The potential signal peptide and transmembrane domain were predicted with SignalP (http://www.cbs.dtu.dk/services/SignalP/) and TMHMM (https://services.healthtech.dtu.dk/service.php?TMHMM-2.0), respectively. The molecular weight was predicted *via* the ProtParam tool (https://web.expasy.org/protparam/).

### Pathogenic bacteria and challenge

*Streptococcus agalactiae* (ZQ0910) was isolated from Nile tilapia and kept in the laboratory (28). The preserved strain was first cultured in a fresh brain–heart infusion liquid medium at 28°C overnight at 80 rpm, then collected *via* centrifugation (4000 g, 5 min), washed thrice in phosphate-buffered saline (PBS) and resuspended in PBS. Subsequently, 50 fish were peritoneally injected with 100 µL/fish of *S. agalactiae* (10^8^ CFU/mL). Every three fish were sacrificed at five different time points (0 h, 6 h, 12 h, 24 h, and 48 h) post challenge. The five tissues such as brain, head kidney, intestine, liver, and spleen, most affected by *S. agalactiae* were collected, and then RNA extraction and cDNA synthesis were immediately executed as mentioned above.

### Quantitative real-time PCR (qRT-PCR)

The tissue distribution of serotonergic marker transcripts in healthy Nile tilapia and the expression pattern post challenge were performed with TB Green^®^ Premix Ex *Taq™* II (Tli RNaseH Plus) (TaKaRa, Dalian, China) and QuantStudio 6 and 7 Flex Real-Time PCR Systems (Thermo Fisher Scientific, Waltham, USA), following the manufacturers’ instructions. *β-actin*, *GAPDH*, and *EF1a* (**Table S1**; **Figure S1**) were used as reference genes. The relative expression levels were calculated following Vandesompele’s (29) and Hellemans’s (30) methods. All reactions were performed with three sample replicates and three technical replicates.

### Western blot

Western blot were used to measure the tissue distribution and relative expression of healthy Nile tilapia HTR1A (*Oreochromis niloticus* HTR1A, On-HTR1A) and On-TPH2 proteins. Total protein from the brain, gills, head kidney, intestine, liver, muscle, skin, and spleen tissues was isolated *via* a protein extraction kit (BC3710, Solarbio, Beijing, China). For each sample, 20 μg total protein was loaded on 12% SDS-PAGE and transferred to a PVDF membrane (IPVH00010, Merck, Darmstadt, Germany).

The membrane was blocked with QuickBlock™ Blocking Buffer for Western Blot (Beyotime, Shanghai, China) at 25°C for 15 min. Membranes were then incubated with primary antibodies, anti-HTR1A (E-AB-12217, Elabscience, Wuhan, China, recognizes the immunogen sequence 101–153 aa of human HTR1A that is 94% (50/53) consistent with Nile tilapia HTR1A) and anti-TPH2 (bs-8729R, Bioss, Beijing, China, recognizes the immunogen sequence 201–300 aa of human TPH2 that is 88% (88/100) consistent with Nile tilapia TPH2). Antibodies were diluted at a ratio of 1:1000 in QuickBlock™ Primary Antibody Dilution Buffer for Western Blot (Beyotime, Shanghai, China) and incubated at 25°C for 1 h. Additionally, the antibody anti-GAPDH (HRP conjugated) (BM3896, BOSTER, Wuhan, China) was utilized to detect the relative expression level of the reference protein, GAPDH, as a control.

Next, the former two membranes were washed three times in TBST (TBS+ 0.1% Tween 20) and incubated with secondary antibody HRP-labeled goat anti-rabbit IgG (H + L) (A0208, Beyotime, Shanghai, China), diluted at a ratio of 1:2000 in QuickBlock™ Secondary Antibody Dilution Buffer for Western Blot (Beyotime, Shanghai, China) at 25°C for 30 min. Finally, all membranes were washed three times in TBST, and the antigen–antibody complexes were detected *via* the enhanced chemiluminescence method (P0018S, Beyotime, Shanghai, China).

### Histological observation

Brain tissues were collected from healthy Nile tilapia and fixed in Dietrich’s fixative for over 20 h. Then, the samples were dehydrated through a graded alcohol series (70%, 85%, 95%, and 100%), cleared in xylene, and embedded in paraffin wax.

Serial sections 5 μm in thickness were stained with a haematoxylin and eosin (H&E) staining kit (C0105S, Beyotime, Shanghai, China), following the manufacturer’s protocols. The sections were observed, photographed, and spliced using a ZEISS Axioscope 5 microscope (Zeiss, Jena, Germany).

### Immunofluorescence (IF)

The serial brain sections from healthy fish containing typical brain structures were collected on the basis of the H&E results. The sections were first rehydrated with PBT (PBS + 0.1% Tween 20) and then incubated with 3% H_2_O_2_ to inactivate endogenous peroxidase, and with EDTA antigen retrieval solution (P0085, Beyotime, Shanghai, China) at 95°C to retrieve antigens. Subsequently, samples were blocked with QuickBlock™ Blocking Buffer for Immunol Staining (Beyotime, Shanghai, China) at room temperature for 15 min and washed three times in PBT. Then, the primary antibody, anti-HTR1A was added and incubated at 25°C for 1.5 h. Next, the samples were washed three times in PBT and incubated with the secondary antibody HRP-labeled goat anti-rabbit IgG (H + L) at 25°C for 1 h. After washing three times in PBT, a Tyramine signal amplification kit (Y6085L, UElandy, Shanghai, China) was applied to label the HTR1A with YF^®^488, following the manufacturer’s protocols.

Subsequently, HRP was inactivated following microwave boiling and then incubated with the anti-serotonin antibody (S5545, Sigma, Darmstadt, Germany), diluted at a ratio of 1:2000, and then incubated with the secondary antibody as mentioned above. Finally, another Tyramine signal amplification kit (Y6088L, UElandy, Shanghai, China) was used to label serotonin with YF^®^555. The samples were observed and photographed as mentioned above.

### Nile tilapia head kidney leukocyte (HKL) preparation

Nile tilapia HKLs were prepared as our previously described (31). Briefly, three healthy fish were sacrificed as mentioned above and then the head kidney of each fish was separated, cut, and sieved through a 40 μm stainless nylon mesh (Greiner Bio-OneGmbH, Frickenhausen, Germany) for collecting the cells which were suspended in Leibovitz’s L-15 Medium (Thermo Fisher Scientific, Waltham, USA). The cells were very gently added to 34%/51% percoll (Solarbio, Beijing, China) and centrifuged at 400 g for 40 min. Then, the leukocytes between the 34% percoll layer and the 51% percoll layer were carefully aspirated. The leukocytes were gently washed with PBS, centrifuged at 500 g for 10 min for collection, and then resuspended and cultured in Leibovitz’s L-15 Medium (Thermo Fisher Scientific, Waltham, USA). A cell counting plate was utilized to count the cells from each fish.

### Nile tilapia HKL single-cell RNA-seq sequencing and cell clustering

The single-cell RNA-Seq (scRNA-Seq) data of Nile tilapia HKL was reported in our previous publication (31). Succinctly, the two samples [Control (PBS stimulated) and Treated (Poly I:C stimulated), each sample was mixed by HKLs of five fish] were prepared for scRNA-seq (10× Chromium platform), and then, 24,062 cells were sequenced and analyzed. The sequencing reads were aligned to the Nile tilapia genome (GCF_001858045.2), and the data quality was evaluated *via* the 10× Genomics official analysis software Cell Ranger. The corrected barcodes and unique molecular identifiers (UMIs) were filtered, and the low-quality cells were removed. Finally, Seurat software normalized the expression of data (31).

However, the subsequent cell clustering and further analysis were slightly adjusted for subsequent experiments *via* an online tool (https://demo.omicsmart.com/10X/home.html#/) in this study. The upper limit value of UMI per cell was adjusted from 30000 to 15000 to filter the error caused by more than one cell captured by the same Gel Beads-In-Emulsions more strictly, especially the smaller fish immune cells (**Figure S3**). After cell subgroup classification and visualization by tSNE (t-distributed stochastic neighbor embedding) nonlinear clustering method, 25 original clusters were detected.

Based on our previous research (31), numerous additional marker genes were applied to identify further subgroups of hematopoietic stem cell/common lymphoid progenitors and cytotoxic T cells. However, monocytes (Mo)/macrophages (MΦ) and granulocytes were not distinguished in this study. A series of neuron marker genes were also involved and analyzed for the subsequent experiment.

### Expression analysis of serotonergic markers from Nile tilapia HKL scRNA-Seq and sequence analysis of *HTR6*

The expression levels of serotonergic markers from Nile tilapia HKL scRNA-Seq were also analyzed *via* the online tool (https://demo.omicsmart.com/10X/home.html#/). We found that these genes were rarely expressed among each immune cell subgroup, except *HTR6*, which was highly and somewhat equally expressed.

Hence, the multiple sequence alignment of *HTR6* raw reads from Nile tilapia HKL scRNA-Seq (**Data S2**) was conducted *via* DNAMAN software (version 7.0). The common sequence among them was first compared with the reference genome (GCF_001858045.2) used in scRNA-Seq data analysis *via* BioEdit (version 7.0.9) and completely matched to the 5′UTR (untranslated region) of *HTR6* (XM_025902001.1).

This sequence was further compared with the lasted Nile tilapia genome on ensembl (http://ftp.ensembl.org/pub/release-105/fasta/oreochromis_niloticus/) and a completely matched cDNA sequence (ENSONIT00000060259.1) was identified *via* NCBI blastx (https://blast.ncbi.nlm.nih.gov/). Consequently, a presumed gene, *mitochondrial contact site and cristae organizing system subunit 10* (*micos10*), was discovered and then cloned with cDNA of head kidney and specific primers (**Table S1**) *via* PCR, as mentioned above. Besides, a 5′ RACE assay was performed to amplify the 5′UTR sequence of *HTR6*, following the manufacturer’s protocols for the SMARTer RACE 5′/3′ Kit (Clontech, Mountain View, USA).

### RNA-Seq of Nile tilapia HKL

RNA-Seq of Nile tilapia HKL was conducted to further assess the expression levels of serotonergic marker transcripts. Three healthy fish were collected to prepare the HKLs, and then, the total RNA was extracted as mentioned above.

The RNA-Seq library construction and RNA-sequencing were completed by Gene Denovo (Guangzhou, China). Briefly, Oligo-dT beads (Qiagen, Hilden, Germany) were used to enrich the mRNA. Then, the mRNAs were broken into short fragments and reverse-transcribed into cDNA with random primers. Subsequently, the cDNA fragments were extracted, end-repaired, ligated with Illumina sequencing adapters, PCR amplified, and sequenced *via* Illumina HiSeq2500. Finally, the raw reads were filtered to obtain high-quality clean reads by fastp. The paired-end clean reads were then mapped to the reference genome (GCF_001858045.2). The RNA-Seq data were submitted to the NCBI Sequence Read Archive (accession number: PRJNA832749).

In addition, an extra *de novo* assembly conducted with the clean reads was jointly assembled into unigenes using Trinity (version 2.8.4). The gene expression abundances were calculated and normalized to fragment per kilobase of transcript per million mapped reads (FPKM).

### 5-HT function on Nile tilapia HKL response to stimulation assay

To assess the role of 5-HT on regulating the HKL response to stimulation, lipopolysaccharide (LPS, dissolved in Leibovitz’s L-15 Medium, 1 mg/mL) (Beyotime, Shanghai, China) and lipoteichoic acid (LTA, dissolved in Leibovitz’s L-15 Medium, 1 mg/mL) (Shanghai Yuan Ye Bio, Shanghai, China) were applied as stimulation models. Specifically, LPS was used to simulate the conditions of Gram-negative bacterial infection as well as LTA for Gram-positive bacterial infection. 5-HT (IH0350, Solarbio, Beijing, China) was dissolved in Leibovitz’s L-15 Medium at a concentration of 20 mg/mL (91 mM) before use.

Three healthy Nile tilapia were collected, and the HKL of each fish was prepared as mentioned above (working as three biological replicates). HKLs were cultured in 12-well plates at 10^5^ cells per well. LPS and LTA were added to the HKL well to a final concentration of 5 μg/mL, respectively. Besides, the same volume of Leibovitz’s L-15 Medium was added to the control group to simulate a resting state.

5-HT was added to each group at a final concentration of 0 (same volume of Leibovitz’s L-15 Medium replaced), 10 nM, and 10 μM, respectively. Then, the HKLs were collected *via* centrifugation (1500 g, 2 min) at 0 min, 10 min, 30 min, 3 h, and 6 h post addition. RNA was extracted and reverse-transcribed as mentioned above. qRT-PCR was performed to assess the potential functions and molecular mechanisms of 5-HT in regulating immune responses using various related genes. **Table S1** lists all primers.

### HTRs involvement and function on Nile tilapia HKL response to stimulation assay

To further assess the involvement and function of HTRs on regulating HKL response to stimulation, the HTR antagonist BMY7378 (SD9540, Beyotime, Shanghai, China), dissolved in Leibovitz’s L-15 Medium at the concentration of 5 mg/mL (10.9 mM), was prepared.

HKLs from three Nile tilapia were prepared and divided into three groups (control, LPS-stimulating group, and LTA-stimulating group), as mentioned above. Also, the same volume of Leibovitz’s L-15 Medium, 5-HT, BMY7378, or the mixture of 5-HT and BMY7378 was added to each well to the final concentration of 10 μM, respectively. Subsequently, the HKLs were collected, processed, and then analyzed for HTR effects on HKL response to stimulation *via* qRT-PCR.

### Drawings and statistical analysis

Adobe Photoshop CC (San Jose, CA, USA) and Adobe Illustrator (San Jose, CA, USA) were used for drawings and final panel designing. TB tools (version 1.098685) were used for drawing heat maps. All data were presented as means ± standard deviations (SD). The one-way ANOVA with *post hoc* tests (Tukey HSD test) and Student’s t-tests were used to analyze the significant difference *via* Prism software (version 8.0). Different letters and asterisks illustrate statistically significant differences (*p* < 0.05).

## Results

### Characteristics of TPH1, TPH2, SERT, MAO, and HTRs

**Figure 1:**
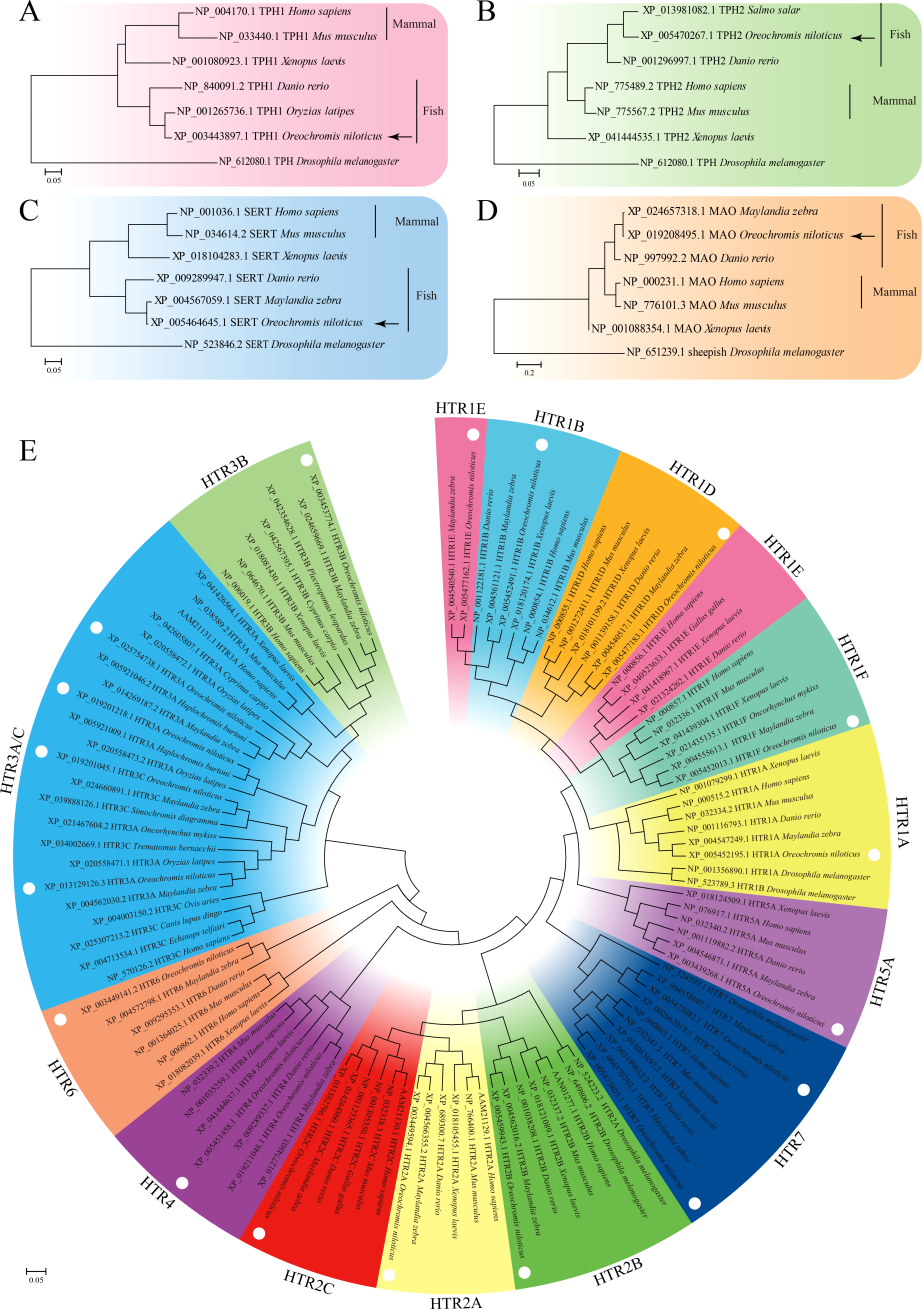
Phylogenetic tree of TPH1 **(A)**, TPH2 **(B)**, SERT **(C)**, MAO **(D)**, and HTRs **(E)** among distinct species constructed using the maximum likelihood (ML) method. The model animal *Drosophila melanogaster* was set as out group.

The open reading frames (ORFs) of *TPH1 (On-TPH1)*, *On-TPH2*, *On-SERT*, *On-MAO*, and *On-HTRs* of *O. niloticus* were cloned, and the resulting multiple sequence alignments indicated that these deduced amino acid sequences contain a series of highly conserved domains (**Data S1**). Phylogenetic analysis showed that these genes initially clustered with other fish lineages and then clustered with amphibians and mammals (**Figure 1**). More than one On-HTR3A, On-HTR4, and On-HTR7 were identified in the Nile tilapia genome and subsequently cloned (**Data S1**).

### Expression characteristics of *TPH1, TPH2, SERT, MAO*, and *HTRs* among different tissues

**Figure 2:**
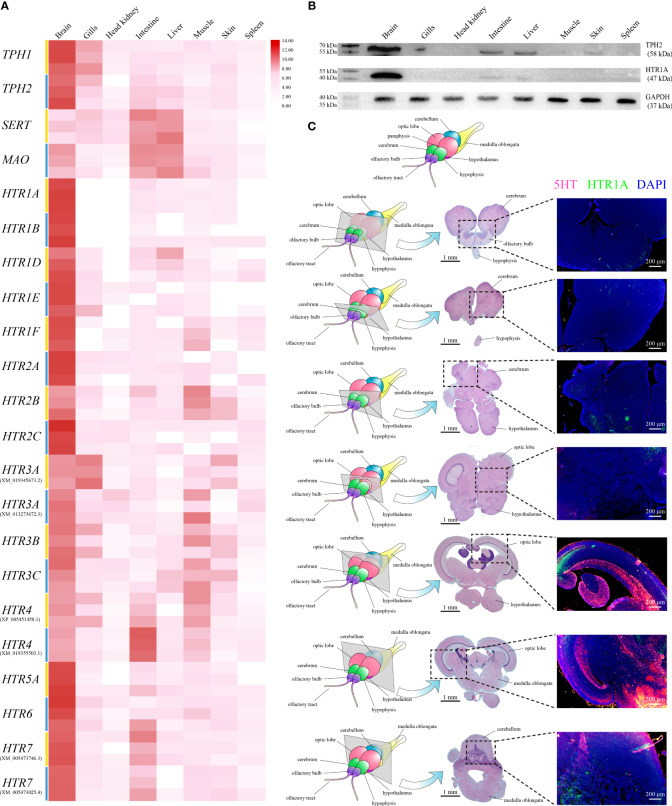
**(A)** Relative expression levels of *On-TPH1*, *On-TPH2*, *On-SERT*, *On-MAO*, and *On-HTRs* mRNA among different tissues in healthy Nile tilapia, assessed *via* qRT-PCR. n = 3. Expression level of each gene in the brain was set as 1000. **(B)** Western blot analysis of On-HTR1A and On-TPH2 in different tissues of healthy Nile tilapia. GAPDH was applied as a reference protein. See **Figure S2** for the complete figures. **(C)** Location of 5-HT and On-HTR1A in the Nile tilapia brain. Top, diagram of Nile tilapia brain morphology. Left column, diagram of sections with typical brain structures in healthy Nile tilapia. Midcolumn, H&E of each selected section. Right column, On-HTR1A (green), 5-HT (red), and nucleus (blue) of each section determined *via* immunofluorescence (IF).

qRT-PCR was applied to assess the relative expression levels of *TPH1, TPH2, SERT, MAO*, and *HTRs* among different tissues in healthy Nile tilapia. The highest *On-TPH1* and *On-TPH2* expression levels were observed in the brain whereas *On-SERT* and *On-MAO* were highly expressed in the intestine and liver. The highest expression of the most *On-HTRs* was also observed in the brain, except *On-HTR3A* (XM_019345673.2) and *HTR4* (XM_019355503.1) which had the highest expression levels in the gills and intestine, respectively. Moreover, the expression of a few *On-HTRs* was not detected in gills, head kidney, liver, or spleen (**Figure 2A**).

The highest levels of On-TPH2 and On-HTR1A proteins were also detected in the brain, whereas no positive band of On-HTR1A was assayed in multiple tissues *via* Western blot (**Figure 2B**). The discernible positive signals of 5-HT and On-HTR1A were both mainly detected in the raphe nuclei, optic lobe, cerebellum, and medulla oblongata of Nile tilapia brain (**Figure 2C**) by IF.

### Involvement of TPH1, TPH2, SERT, MAO, and HTRs during bacterial challenge

**Figure 3:**
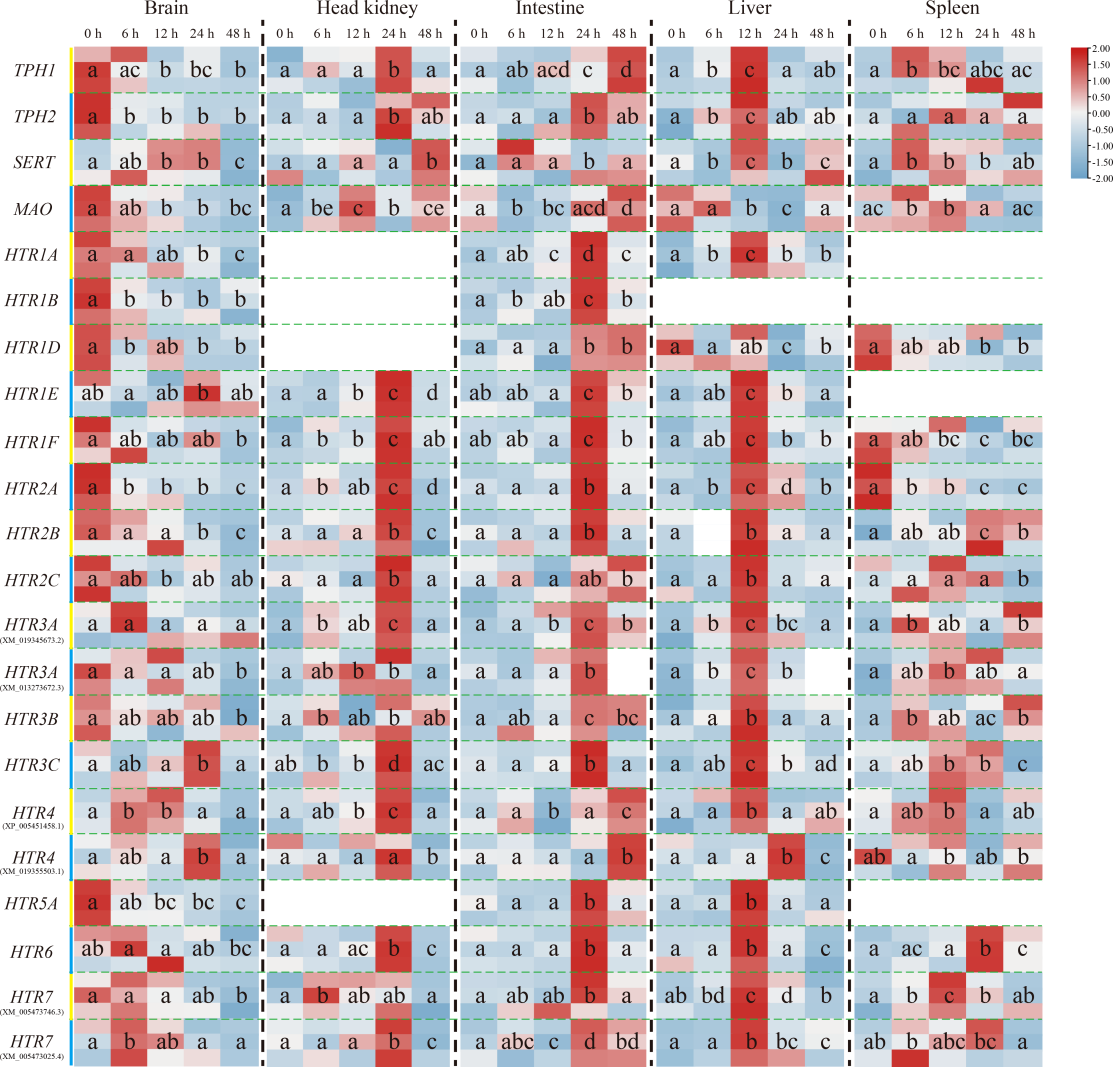
Expression patterns of *On-TPH1*, *On-TPH2*, *On-SERT*, *On-MAO*, and *On-HTRs* mRNA among different tissues (the brain, head kidney, intestine, liver, and spleen) at a series of time points after *S. agalactiae* injection, as detected *via* qRT-PCR. The expression level of these genes at 0 h is set as 1.00 to calculate the relative expression of the other time points. n = 3. The significant difference analysis was using *post hoc* tests (Tukey HSD test) with one-way ANOVA and the different letters indicated significant difference (*p* < 0.05).

The transcriptional levels of the other genes, except *On-SERT, On-HTR1E, On-HTR3C*, and *On-HTR4* genes, mainly decreased in the brain post challenge. Nevertheless, their expression increased as a whole in the head kidney, intestine and liver. The expression peak reached at 24 h after challenge in the head kidney and intestine, whereas earlier (12 h) in the liver. Moreover, in the spleen, *On-TPH1, On-TPH2, On-SERT, On-MAO*, and most On-HTRs were promoted post challenge while the expression levels of *On-HTR1D, On-HTR1F*, and *On-HTR2A* were down regulated (**Figure 3**).

### Nile tilapia HKLs clustered into six subgroups

Differing from our previous research that focused on the nonspecific cytotoxic cell (NCC) subgroups (31), we paid more attention to the distribution characteristics of our 22 serotonergic markers among general and widespread HKL subgroups and then minor adjusted the filter standard (**Figure S3**).

**Figure 4:**
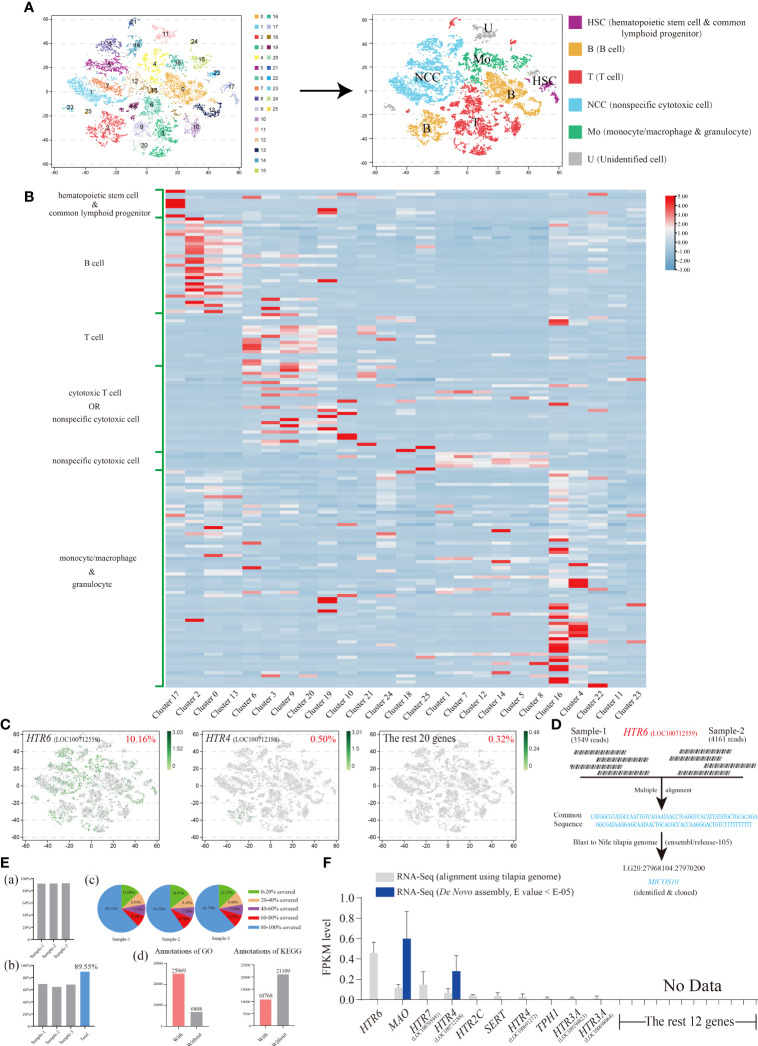
**(A)** The visualization of Nile tilapia HKL population *via* tSNE nonlinear clustering. Left, original classification of Nile tilapia HKL population. Right, identification of six cell subpopulations based on marker genes. **(B)** Heat map of the expression of different types of cell marker genes among each cluster. See **Table S3** for the details of marker genes. **(C)** The tSNE results of the top two genes, *HTR6* and *HTR4*, as well as the remaining 20 genes. The number at the top right of the figure indicates the proportion of positive cells. Each dot in the figure represents a cell. See **Table S4** for the details of marker genes. **(D)** Diagram of analysis process of *HTR6* sequence from scRNA-Seq. **(E)** Basic information of Nile tilapia HKL RNA-Seq aligned *via* reference genome. Proportion of genes mapped to the reference genome **(A)**. Proportion of reference genome genes sequenced in three samples and their union **(B)**. Distribution of genes’ coverage of each sample **(C)**. Number of unigenes annotated *via* GO and KEGG **(D)**. **(F)** Expression level of *On-TPH1*, *On-TPH2*, *On-SERT*, *On-MAO*, and *On-HTRs* in Nile tilapia HKL RNA-Seq aligned *via* reference genome or *de novo* assembled. See **Table S5** for the expression details of candidate genes.

Consequently, 25 original clusters were identified *via* tSNE and then clustered into six cell subgroups based on numerous marker genes (**Figure 4A** and **Figure S4**; **Table S3**). These included hematopoietic stem cells (HSC)/common lymphoid progenitors, B cells, T cells, cytotoxic T cells/NCC, NCCs, and monocytes (Mo)/macrophages (MΦ)/granulocytes (**Figures 4A, B**). Six marker genes representing neurons were also detected, and no obvious expression was observed among HKLs (**Figure S4**; **Table S3**).

### Expression characteristics of *TPH1, TPH2, SERT, MAO*, and *HTRs* in Nile tilapia HKL scRNA-Seq

The expression characteristics of 22 serotonergic markers among HKL were displayed visually *via* tSNE. Meanwhile, 10 of them were not detectable (**Table S4**). Only one gene, *HTR6*, was expressed in more than 1% of cells. In fact, the proportion of *HTR6* positive cells was over 10% and almost equally distributed among each subgroup of HKL (**Figure 4C**).

To further confirm this result, the raw reads (**Data S2**) of *HTR6* in scRNA-Seq were assessed and the common sequence of raw reads matched to the −1700 region of the 5′ UTR of *HTR6* (XM_025902001.1), using the reference genome (GCF_001858045.2). However, this was unlikely due to the scRNA-Seq conducted *via* poly(A). This sequence was then compared with the last Nile tilapia genome on ensemble and matched completely to the gene, *MICSO10*, which was subsequently cloned (**Figure 4D**; **Figure S5**). A 5′ RACE assay of *HTR6* was performed, and only a 25 bp length of the 5′UTR of *HTR6* was amplified (result not shown).

### Expression characteristics of *TPH1, TPH2, SERT, MAO*, and *HTRs* in Nile tilapia HKL RNA-Seq

To evaluate the overall expression characteristics of 22 serotonergic markers in Nile tilapia HKL, three cDNA libraries were constructed and sequenced using the Illumina deep-sequencing platform. A total of 161.01 Mb raw reads were generated, and 160.67 Mb clean reads were obtained with an average Q30 of 92.84% and 92.99%, respectively. Then the high-quality clean reads were aligned using a reference genome and the total mapping rates over 91.7% (**Figure 4E**; **Figure S6, S7**) or assembled *de novo.*

Like the scRNA-Seq results, nearly the same 10 genes of 22 serotonergic markers were detected in the transcriptome profile, aligned using the same reference genome. HTR6 showed the highest expression once again (**Figure 4F**; **Table S5**). However, in the *de novo* assembled profile, only two genes were detected (**Figure 4F**; **Table S5**).

### Effects of 5-HT function on Nile tilapia HKL response to stimulation

**Figure 5:**
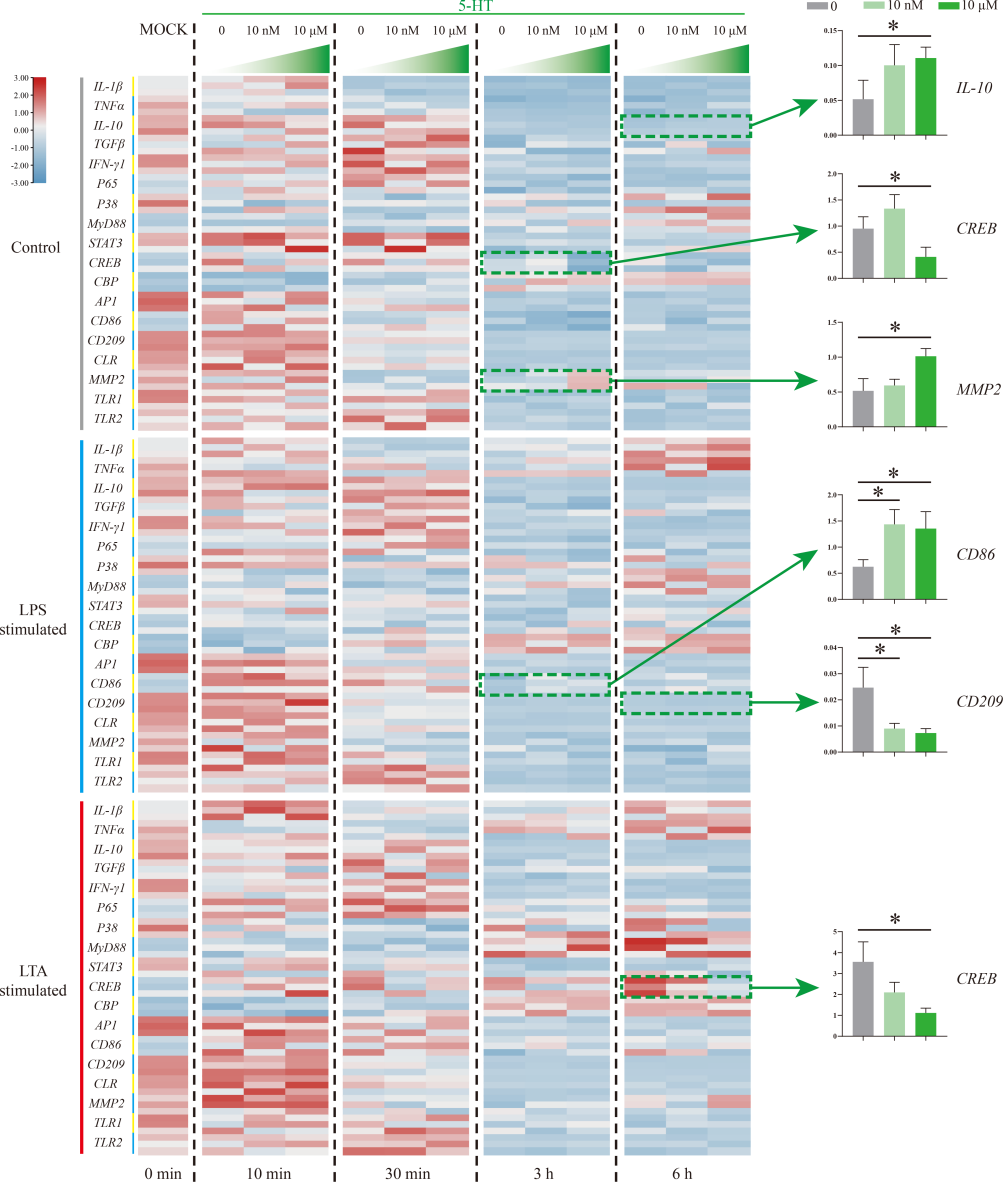
The expression patterns of numerous immune-related genes detected *via* qRT-PCR at different time points among three model groups with different concentrations of 5-HT treatment. n = 3. The significant difference analysis at each time point was performed *via* student’s t-test (compared in pairs) and the all significant different results were further displayed by the histograms on the right as well as indicated with asterisks (*p* < 0.05). See **Table S2** for the expression details of candidate genes.

5-HT function on Nile tilapia HKL response to stimulation was further evaluated by detecting 18 immune-related genes *via* qRT-PCR (**Figure 5**). In the control group, three genes (*IL-10*, *CREB*, and *MMP2*) were significantly induced by 5-HT (10 μM) at 3 and 6 h. Specifically, *IL-10* and *MMP2* were promoted but *CREB* was suppressed. However, the expressions of *CD86* and *CD209* in LPS simulation group were significantly up-regulated and down-regulated, respectively. Moreover, only one gene (*CREB*) in LTA simulation group was remarkably suppressed by 5-HT.

### Effects of HTR involvement and function on Nile tilapia HKL response to stimulation

**Figure 6:**
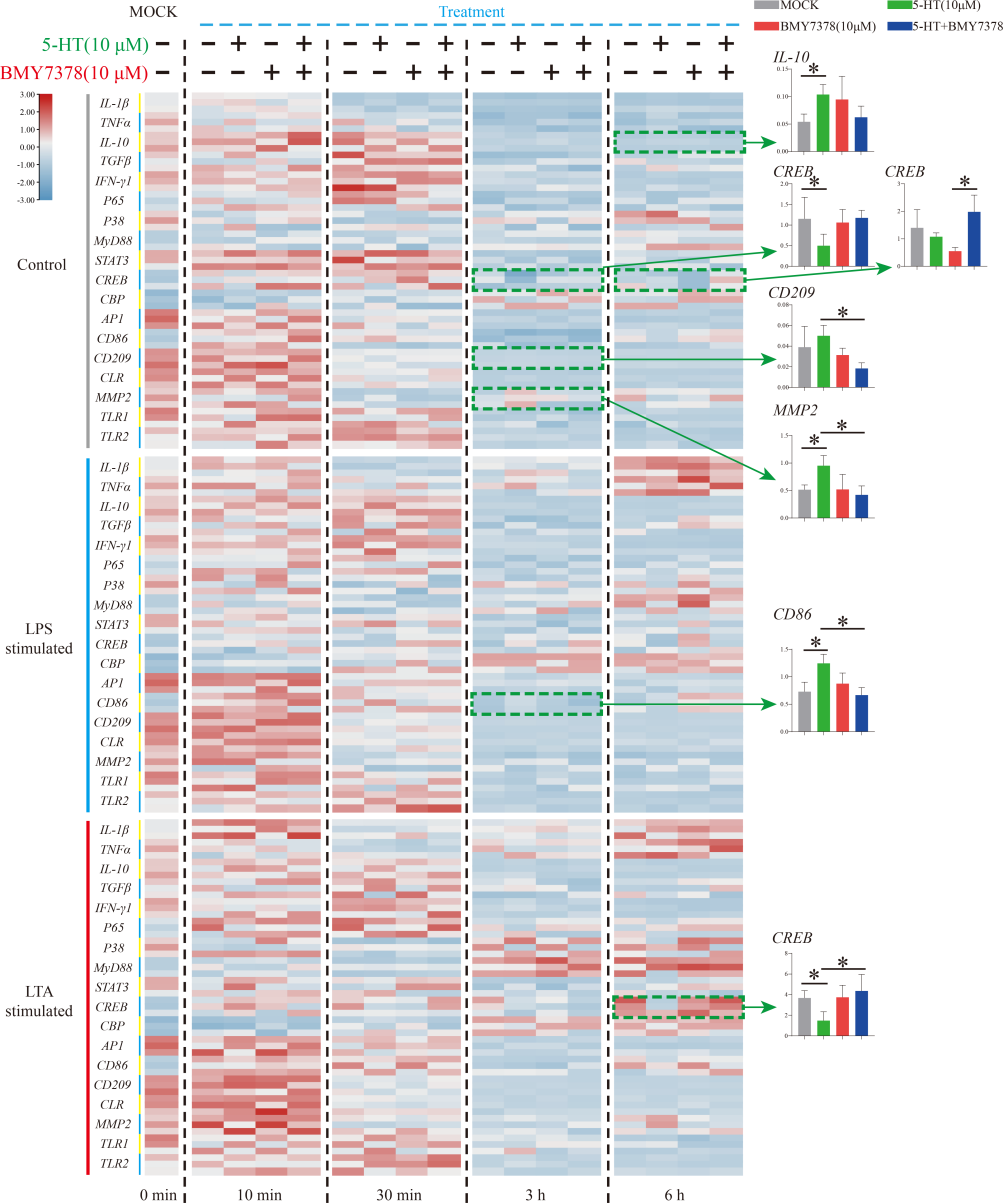
The expression patterns of numerous immune-related genes detected *via* qRT-PCR at different time points among three model groups with 5-HT or/and BMY7378 treatment. n = 3. The significant difference analysis at each time point was performed *via* student’s t-test (compared in pairs) and the all significant different results were further displayed by the histograms on the right as well as indicated with asterisks (*p* < 0.05). See **Table S2** for the expression details of candidate genes.

To further verify the molecular mechanism of the weak modulation of 5-HT on Nile tilapia HKL response to stimulation, an HTR antagonist, BMY7378, was applied (**Figure 6**). During resting status, the significant increase of *IL-10* (6 h) and decrease of *CREB* (3 h) first induced by 5-HT was not modulated after BMY7378 addition. The reduced expression of *CREB* (6 h) after BMY7378 addition was remarkably up-regulated with 5-HT and BMY7378 co-treatment. Additionally, the enhanced expression of *CD209* (3 h) after 5-HT addition was significantly down-regulated with 5-HT and BMY7378 co-treatment. Nevertheless, the significant increase of *MMP2* (3 h) induced by 5-HT was notably suppressed after BMY7378 addition. In the simulation group, the significantly up-regulated *CD86* (3 h, LPS simulation) and down-regulated *CREB* (6 h, LTA simulation) induced by 5-HT were also reversed after BMY7378 addition.

## Discussion

Serotonin was first isolated in 1948 (32) and successively found among all bilateral animals (3, 5, 33). As a neurotransmitter, serotonin is involved not only in neurobiological functions but also in the complex influences on immunity. Nevertheless, the neuroimmune network mediated by serotonin and HTRs has not been widely studied in other vertebrates, such as fish, which constitute the most diverse vertebrate taxa (16). It is considered that CNS organization among vertebrates is structured consistently (15), whereas immune systems vary greatly between fish and mammals (34), further comprehension of fish immune system composition and mechanisms could help us to understand the evolution of the immune system. Perhaps some distinct mechanisms may have evolved in fish to reduce the risk of infectious diseases in aquaculture.

In the present study, 22 serotonergic markers including TPH1&2, SERT, MAO, and 18 HTRs of Nile tilapia were cloned and analyzed, the inferred proteins of these serotonergic markers share high similarity (approximately 80%) with other fish lineages and mammals, implying the significance of the serotonin system during fish evolution, such as modulating the reproductive system at multiple levels *via* the hypothalamo-pituitary-gonadal axis (14), promoting neuron development (22), and sleep (21). Furthermore, the tissue distribution of serotonergic markers at both mRNA and protein levels was evaluated to verification the conservative roles of the serotonin system in Nile tilapia. Similar to other fish, these molecules were mainly expressed in the brain. More specifically, the major regions of serotonin expression were the raphe nuclei and optic lobe, whereas HTR1A was mainly expressed at the periventricular gray zone (16, 19, 35, 36).

After *S. agalactiae* challenge, qRT-PCR results showed that serotonergic marker expression was sharply induced in all test tissues, implying their involvements during bacterial infection in Nile tilapia. Specifically, their expression levels were mainly suppressed in the brain while mainly promoted in rest organs including head kidney, intestine, liver, and spleen. Based on our previous research (37) and Yi’s (24) & Cao’ s studies (27), various Nile tilapia organs rapidly respond to *S. agalactiae* infection. The peritoneal injection usually leads to a first response occurring in the peripheral immune organs such as the head kidney and spleen and a hysteretic response in the brain. Additionally, work from the Lu’ group also indicated that *S. agalactiae* infection affects the swimming behavior of Nile tilapia and the serotonin level in the brain, intestine, and stomach were significantly reduced post challenge (24).

Furthermore, based on our previous work (31), a slightly adjusted filtering threshold value-directed scRNA-Seq reanalysis was conducted, and resulting in a more rigorous expression profile. This profile is clustered into more immune cell subgroups, including almost every known type of fish immune cells. These results support the conclusion that the fish head kidney is character of hematopoiesis previously observed at histological levels (38, 39). However, we did not identify any obvious expression of serotonergic markers in scRNA-Seq or RNA-Seq levels (assembled *de novo*). Similarly, these genes were not detected in the RNA-Seq profiles of other fish head kidneys (40, 41). Totally differences from the situation in human is that immune cells variedly and broadly express serotonergic markers, such as Mo/MΦ express HTR1A, 1E, 2A, 3A, 4, and 7 while B cells express 1A, 2A, 3, and 7, as well as nearly all immune cells express at least one serotonin component (1, 2).

To further explore the roles of serotonin on Nile tilapia immune cells, 5-HT and an HTR antagonist were added into HKLs during different stimulation models. However, few noteworthy results were observed after the assessment of their regulation roles on 18 immune-related genes, whereas most of them were well reported to have a distinct interaction with serotonin *via* HTRs in mammals (1–4). Specifically, only three genes (*IL-10, MMP2*, and *CD86*) were promoted and two genes (*CREB* and *CD209*) were suppressed after serotonin treatment. Moreover, three of them (*MMP2*, *CD86*, and *CREB*) were reversely influenced by the HTR1A antagonist. CD86 (B7-2) was a well-known costimulatory molecule, constitutively expressed in macrophages, dendritic cells, B cells, and any other antigen-presenting cells and was necessary for T cell activation (42, 43). In this study, a consistent result to situations in mammals was observed in that serotonin stimulated *CD86* expression in Nile tilapia immune cells. This is typically caused by an increase in intracellular cAMP levels in mammals (4, 44–46). Another contradictory result was observed that *MMP2* was induced under the resting state. However, the direct relationship of MMP2 involvement in the inflammatory response and serotonin system have not been well studied (47, 48). Meanwhile, the decomposition production of serotonin would induce reactive oxygen species and then promote MMP2 activation (49). Additionally, there are multiple mediation effects of serotonin on CREB phosphorylation and then downstream genes *via* different HTRs in mice and humans (1, 4, 44, 50, 51). Our results indicated that *CREB* in Nile tilapia immune cells was suppressed by serotonin, usually mediated by HTR2 (44).

Moreover, the mediation of serotonin on immunity is widely reported among invertebrates, including insects (52) and mollusks (53). Researchers suggest that the ancient serotonin system might control the immune system across the animal kingdom (52). However, based on our study, the immunomodulation of serotonin on fish immunity is not efficient enough and ambiguous. Additional studies are warranted to obtain knowledge of the modulation of serotonin on immunity in other animal kingdoms, such as echinoderm and amphibians.

In summary, we identified and characterized 22 conservative homologs of serotonergic markers in Nile tilapia. We also determined that the Nile tilapia serotonin system barely expressed in immune cells and induces its immune response, fully distinct from mammals. This study lays a theoretical foundation for further investigation focusing on fish neuroimmunology and, more specifically, the evolution of the serotonin system in fish.

## Data availability statement

The datasets presented in this study can be found in online repositories. The names of the repository/repositories and accession number(s) can be found in the article/**supplementary material**.

## Ethics statement

The animal study was reviewed and approved by the Ethics Committee of the Guangdong Ocean University (Date: 10 May 2019).

## Author contributions

QL and YH designed the experiments. BJ, ZZ, YXH, ZX and XC performed experiments. QL analyzed the data and wrote the manuscript. XH, JC and JJ reviewed the manuscript. JJ provided funds for this research. All authors contributed to the article and approved the submitted version.

## Funding

This work was supported by National Natural Science Foundation of China (No. 32073006, 32002426), National Key R&D Program of China (No. 2018YFD0900501), Fund of Southern Marine Science and Engineering Guangdong Laboratory (Zhanjiang) (No. ZJW-2019-06).

## Conflict of interest

The authors declare that the research was conducted in the absence of any commercial or financial relationships that could be construed as a potential conflict of interest.

## Publisher’s note

All claims expressed in this article are solely those of the authors and do not necessarily represent those of their affiliated organizations, or those of the publisher, the editors and the reviewers. Any product that may be evaluated in this article, or claim that may be made by its manufacturer, is not guaranteed or endorsed by the publisher.
