# Supplementary material for: Serotonin system is partially involved in immunomodulation of Nile tilapia (Oreochromis niloticus) immune cells
Source: Front Immunol. 2022 Jul 28;13:944388. doi: 10.3389/fimmu.2022.944388 (PMC9366525; doi:10.3389/fimmu.2022.944388)
Supplement: Supplementary file 1 [file DataSheet_1.zip › Supplementary materials/Data S1. Multiple sequence alignment/Captions for multiple sequence alignment (Fig 1-19).docx]

Red box indicates the transmembrane domain existed among vertebrates, green & blue box indicates the transmembrane domain only in fishes (Figure 13 &15).

Red asterisk indicates the 5-HT binding site (Figure 1 & 2).

Green asterisk indicates the iron binding site (Figure 1).
