# Supplementary figures and images for: Serotonin system is partially involved in immunomodulation of Nile tilapia (Oreochromis niloticus) immune cells
Source: Front Immunol. 2022 Jul 28;13:944388. doi: 10.3389/fimmu.2022.944388 (PMC9366525; doi:10.3389/fimmu.2022.944388)

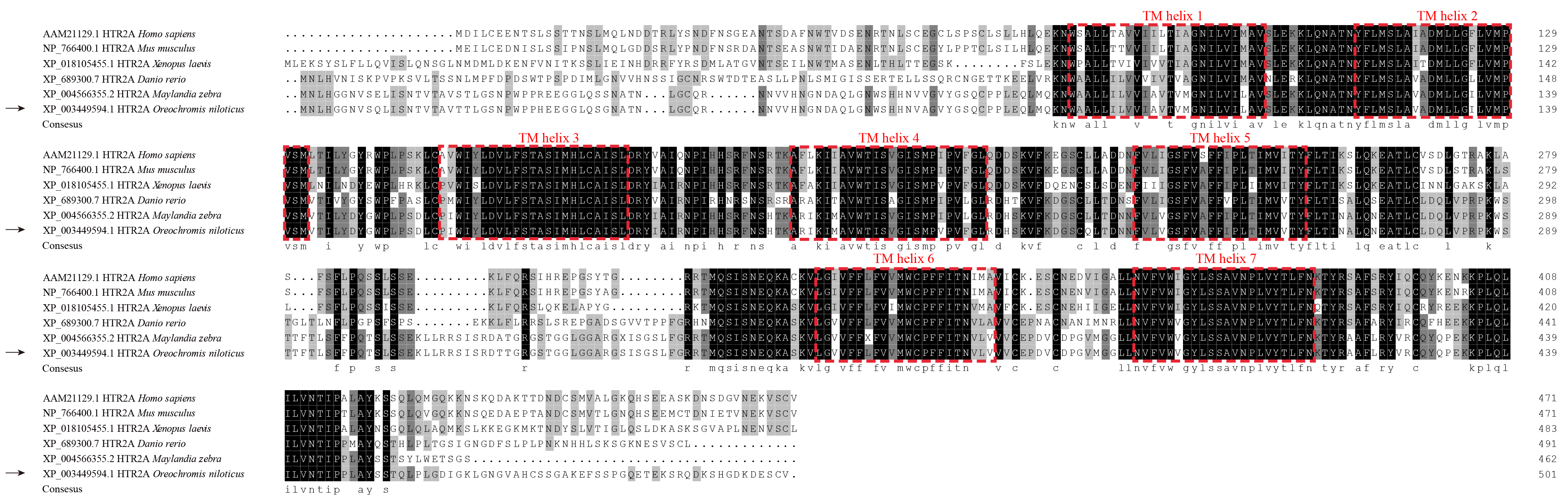

Supplement: Supplementary file 1 [file DataSheet_1.zip › Supplementary materials/Data S1. Multiple sequence alignment/Figure 10-Multiple sequence alignment of HTR2A.tif]

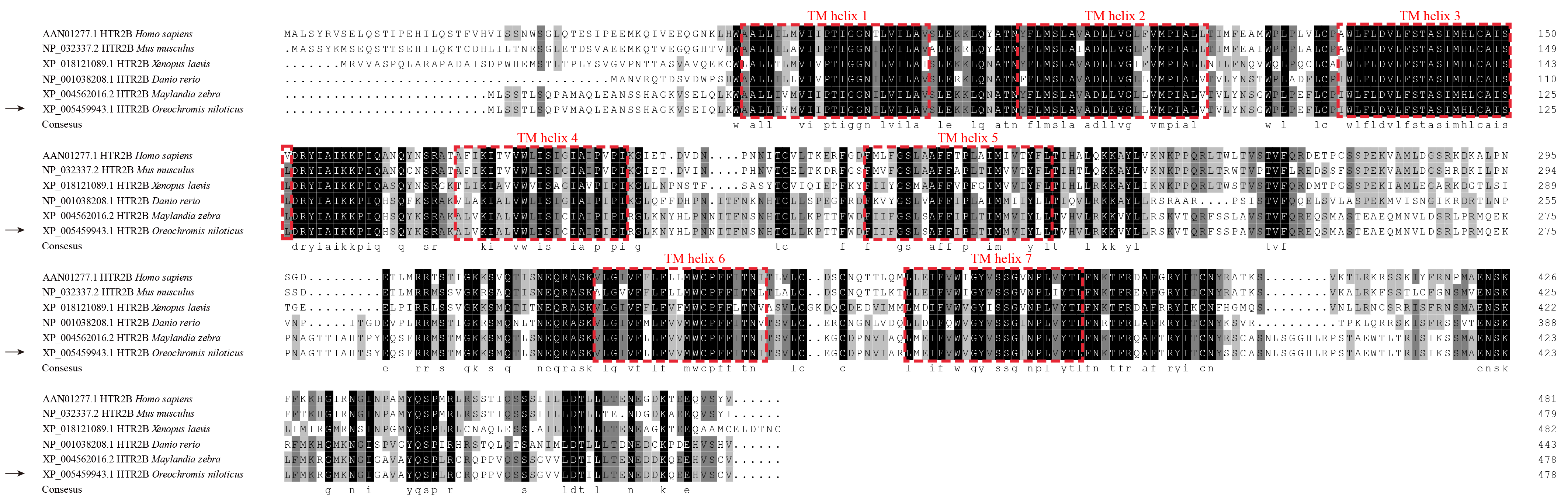

Supplement: Supplementary file 1 [file DataSheet_1.zip › Supplementary materials/Data S1. Multiple sequence alignment/Figure 11-Multiple sequence alignment of HTR2B.tif]

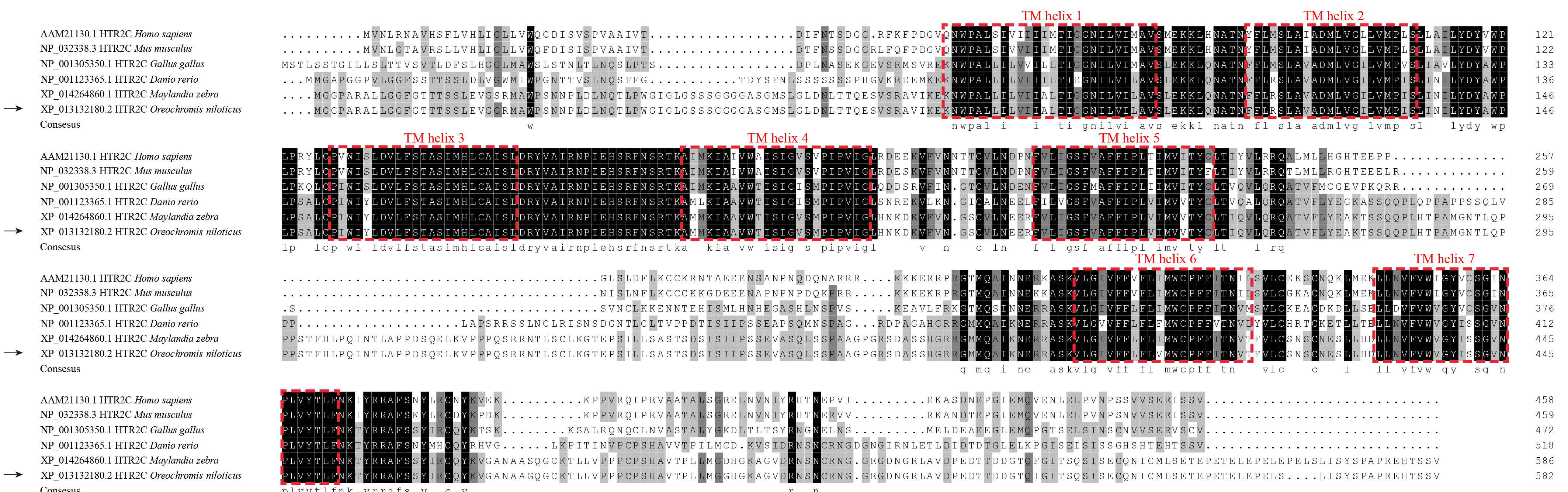

Supplement: Supplementary file 1 [file DataSheet_1.zip › Supplementary materials/Data S1. Multiple sequence alignment/Figure 12-Multiple sequence alignment of HTR2C.tif]

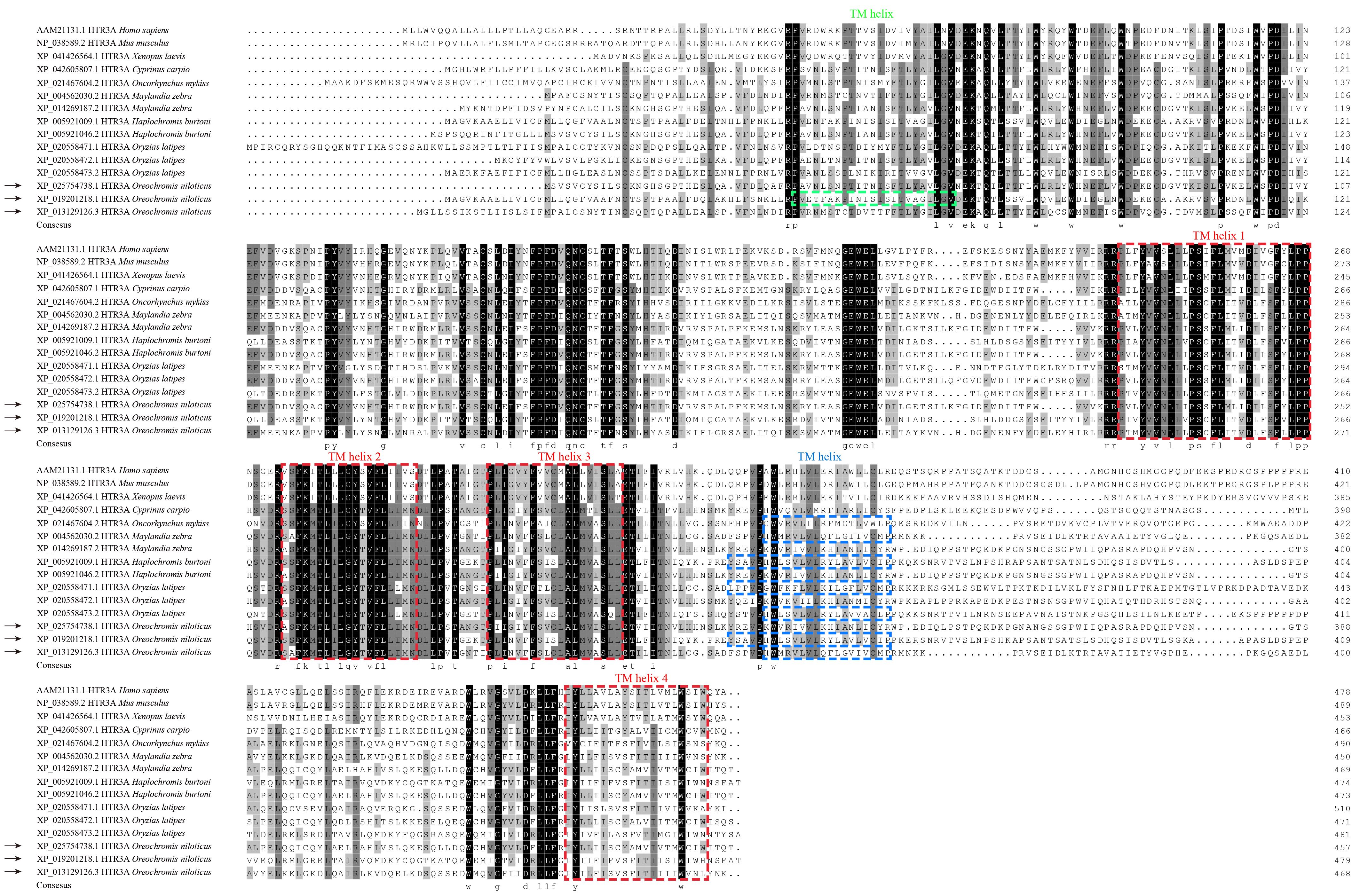

Supplement: Supplementary file 1 [file DataSheet_1.zip › Supplementary materials/Data S1. Multiple sequence alignment/Figure 13-Multiple sequence alignment of HTR3A.tif]

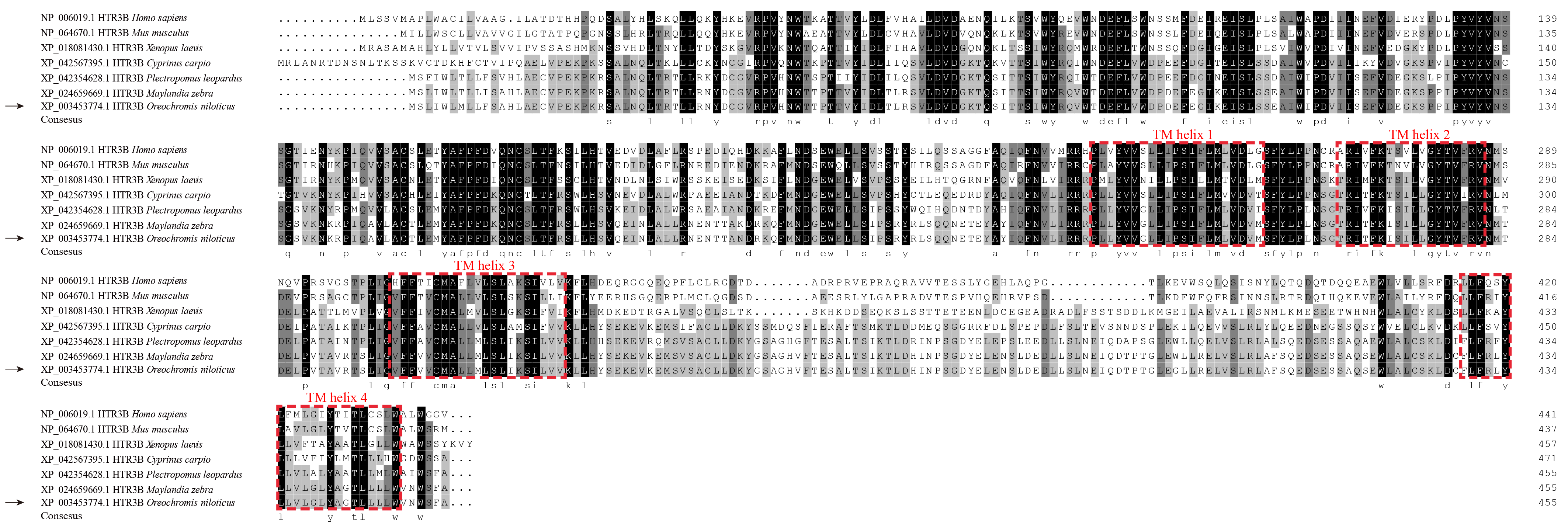

Supplement: Supplementary file 1 [file DataSheet_1.zip › Supplementary materials/Data S1. Multiple sequence alignment/Figure 14-Multiple sequence alignment of HTR3B.tif]

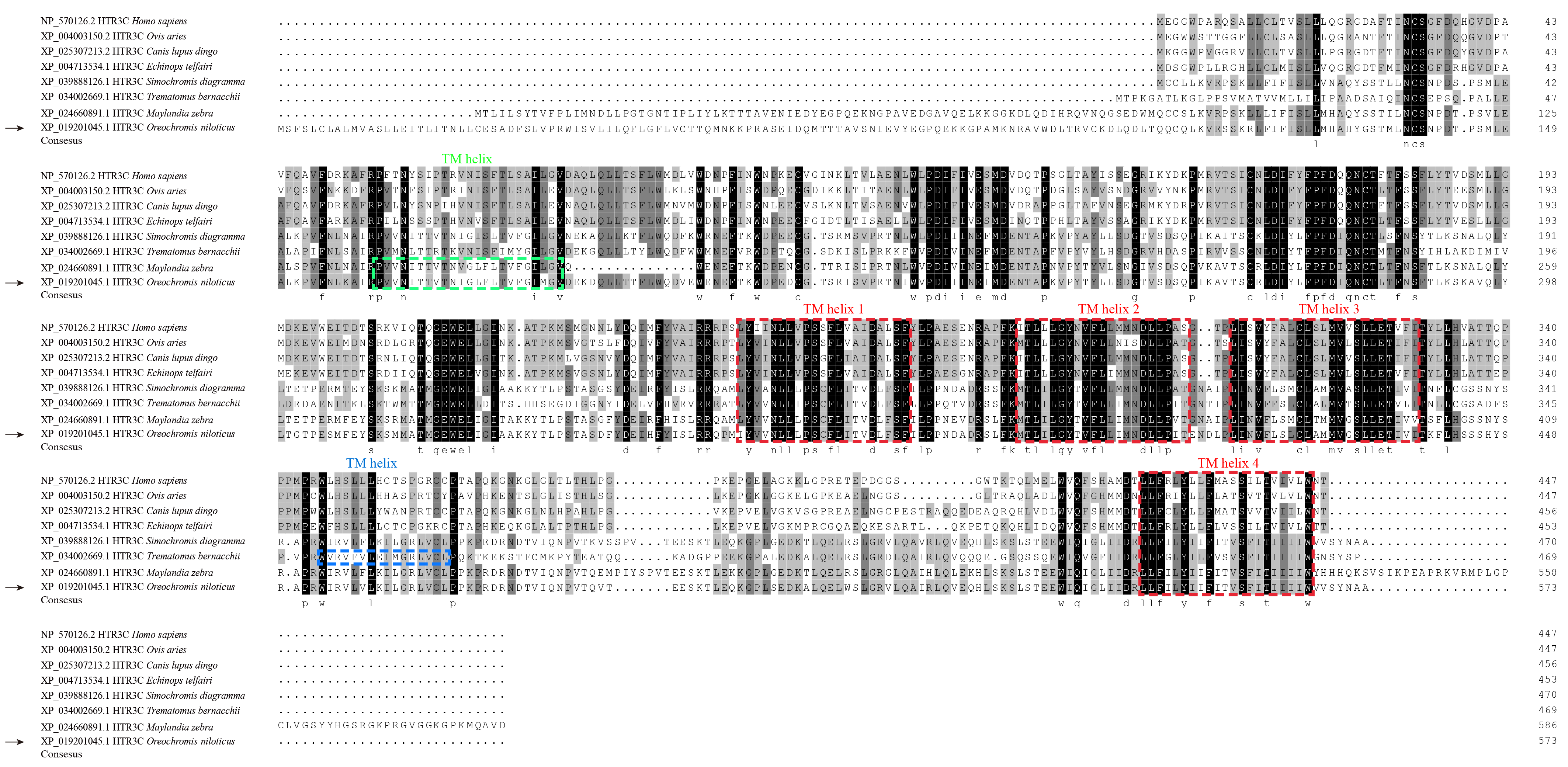

Supplement: Supplementary file 1 [file DataSheet_1.zip › Supplementary materials/Data S1. Multiple sequence alignment/Figure 15-Multiple sequence alignment of HTR3C.tif]

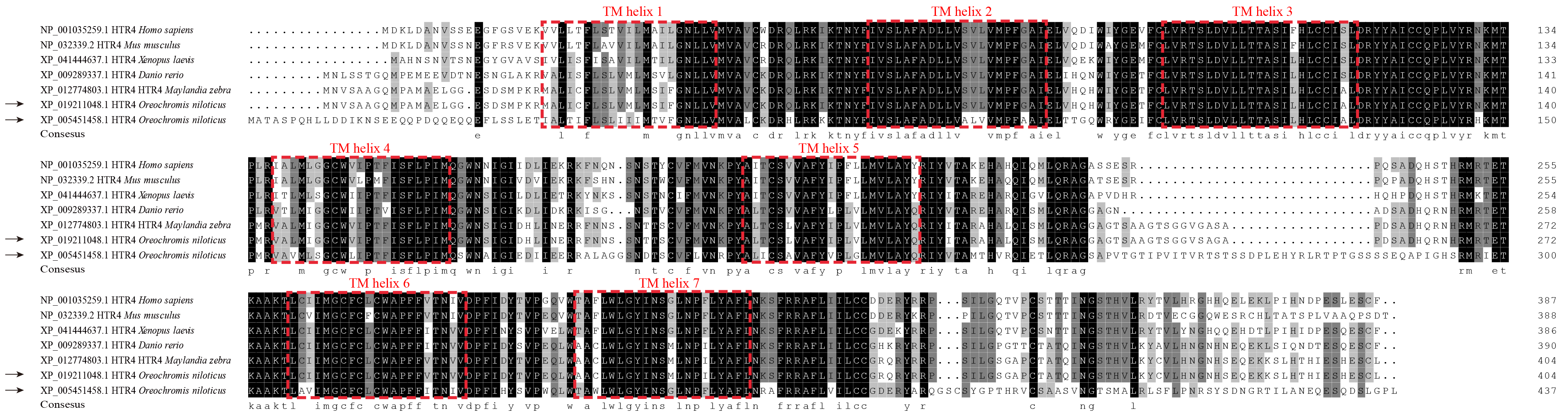

Supplement: Supplementary file 1 [file DataSheet_1.zip › Supplementary materials/Data S1. Multiple sequence alignment/Figure 16-Multiple sequence alignment of HTR4.tif]

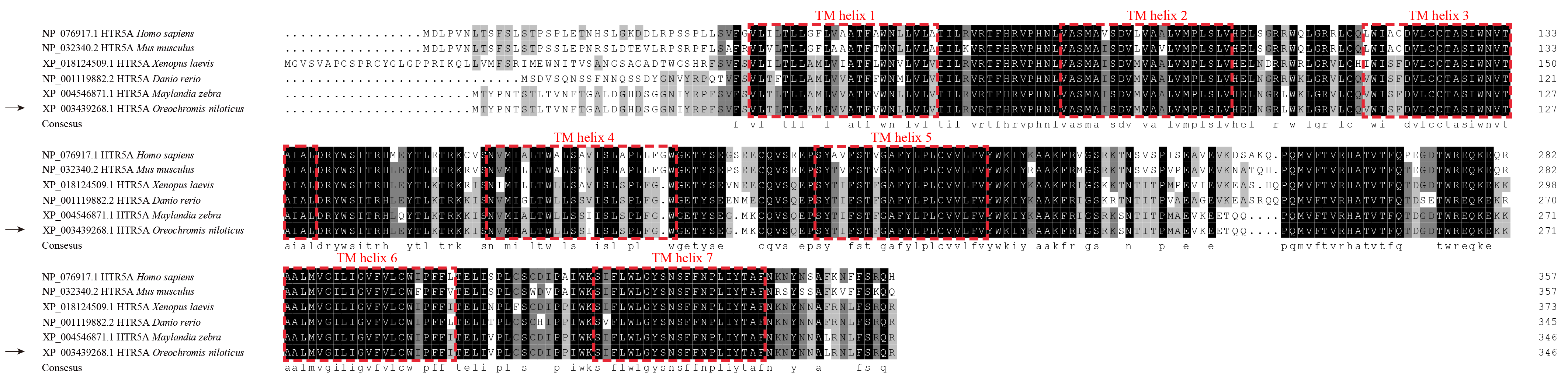

Supplement: Supplementary file 1 [file DataSheet_1.zip › Supplementary materials/Data S1. Multiple sequence alignment/Figure 17-Multiple sequence alignment of HTR5A.tif]

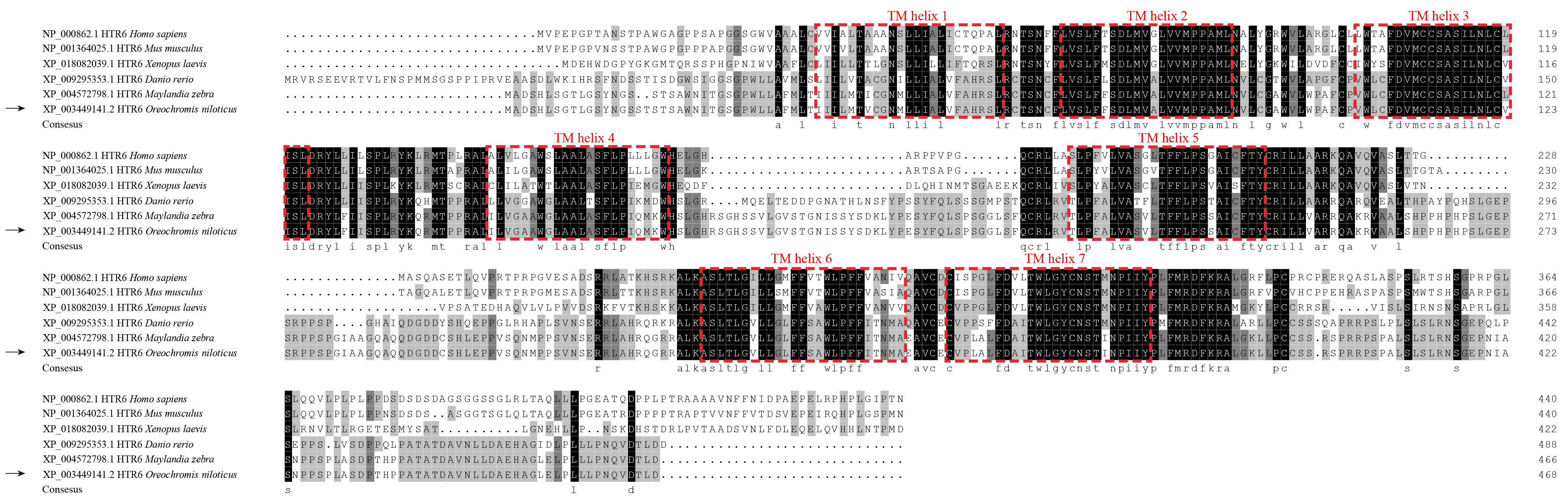

Supplement: Supplementary file 1 [file DataSheet_1.zip › Supplementary materials/Data S1. Multiple sequence alignment/Figure 18-Multiple sequence alignment of HTR6.tif]

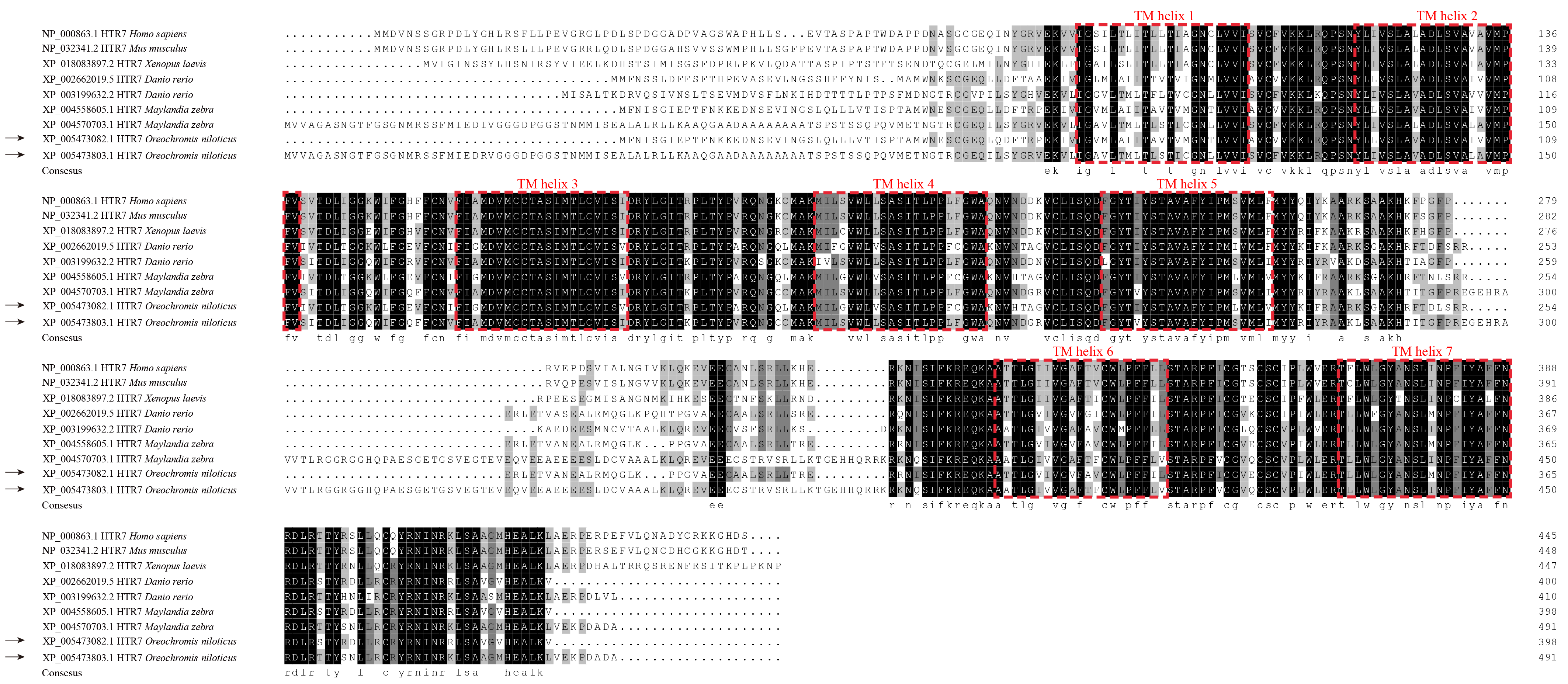

Supplement: Supplementary file 1 [file DataSheet_1.zip › Supplementary materials/Data S1. Multiple sequence alignment/Figure 19-Multiple sequence alignment of HTR7.tif]

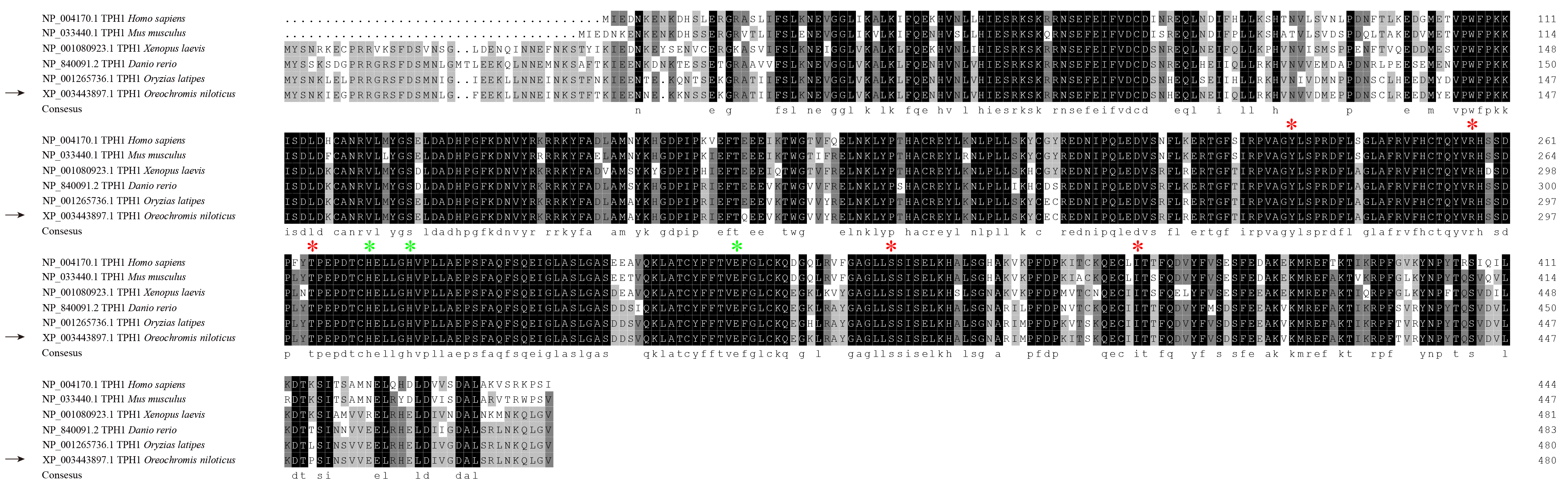

Supplement: Supplementary file 1 [file DataSheet_1.zip › Supplementary materials/Data S1. Multiple sequence alignment/Figure 1-Multiple sequence alignment of TPH1.tif]

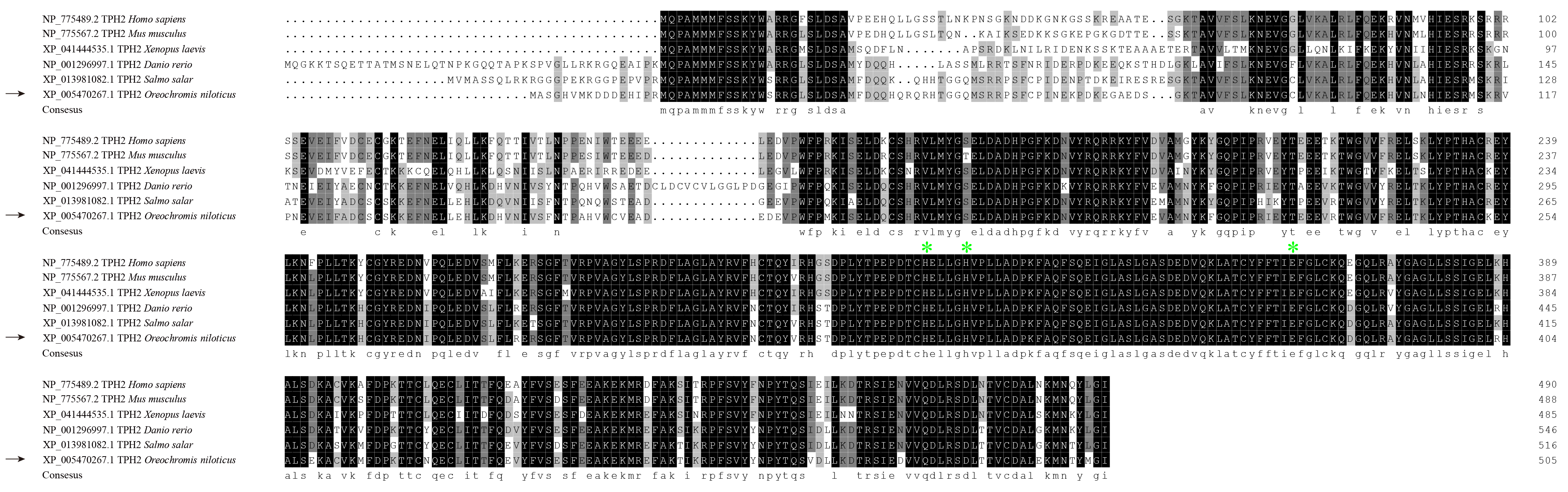

Supplement: Supplementary file 1 [file DataSheet_1.zip › Supplementary materials/Data S1. Multiple sequence alignment/Figure 2-Multiple sequence alignment of TPH2.tif]

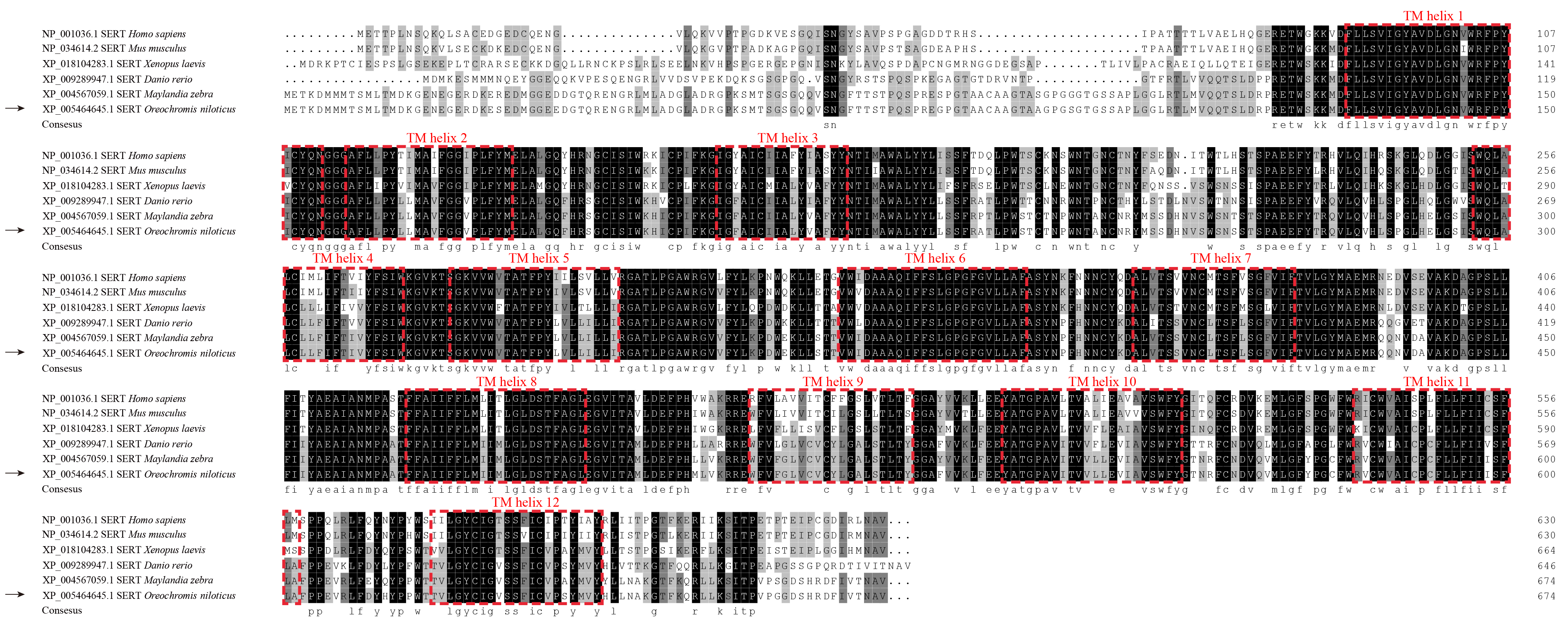

Supplement: Supplementary file 1 [file DataSheet_1.zip › Supplementary materials/Data S1. Multiple sequence alignment/Figure 3-Multiple sequence alignment of SERT.tif]

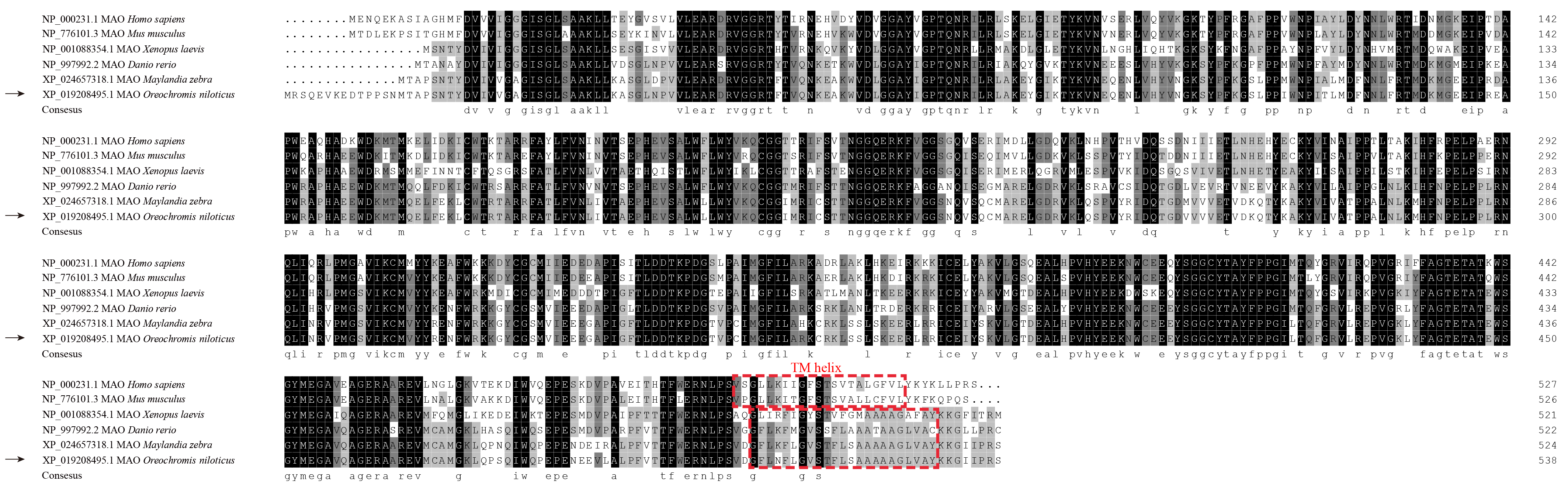

Supplement: Supplementary file 1 [file DataSheet_1.zip › Supplementary materials/Data S1. Multiple sequence alignment/Figure 4-Multiple sequence alignment of MAO.tif]

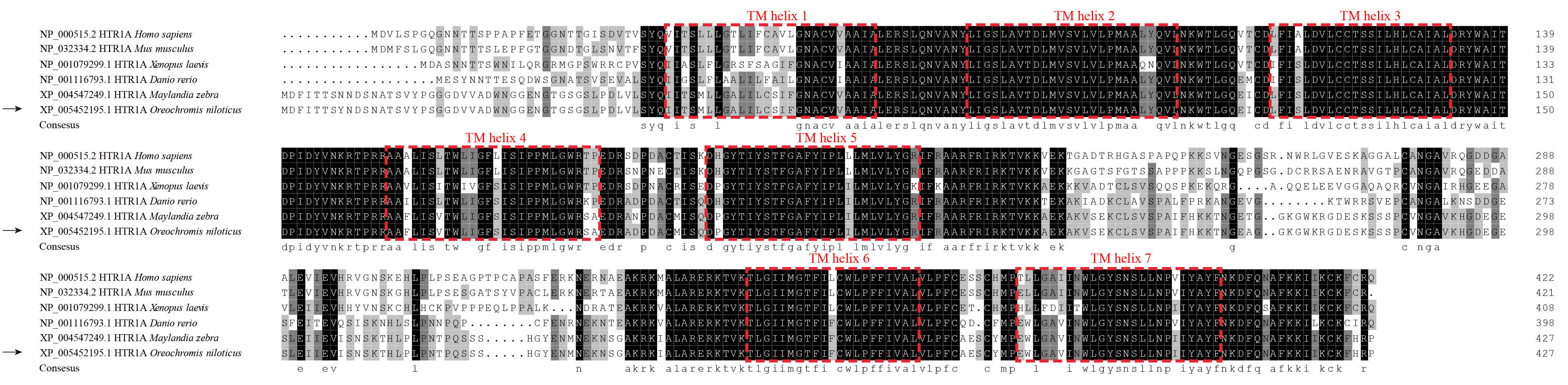

Supplement: Supplementary file 1 [file DataSheet_1.zip › Supplementary materials/Data S1. Multiple sequence alignment/Figure 5-Multiple sequence alignment of HTR1A.tif]

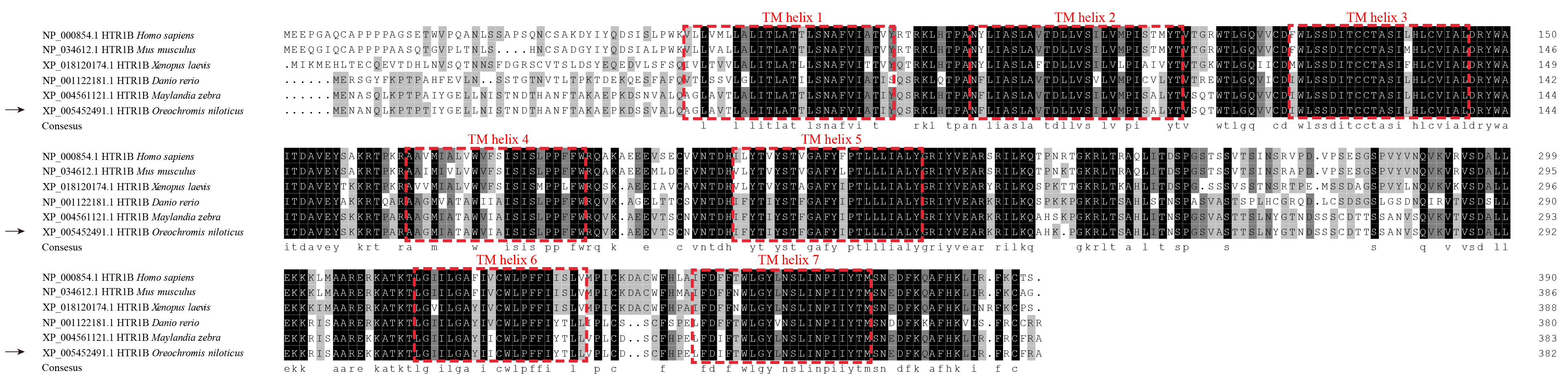

Supplement: Supplementary file 1 [file DataSheet_1.zip › Supplementary materials/Data S1. Multiple sequence alignment/Figure 6-Multiple sequence alignment of HTR1B.tif]

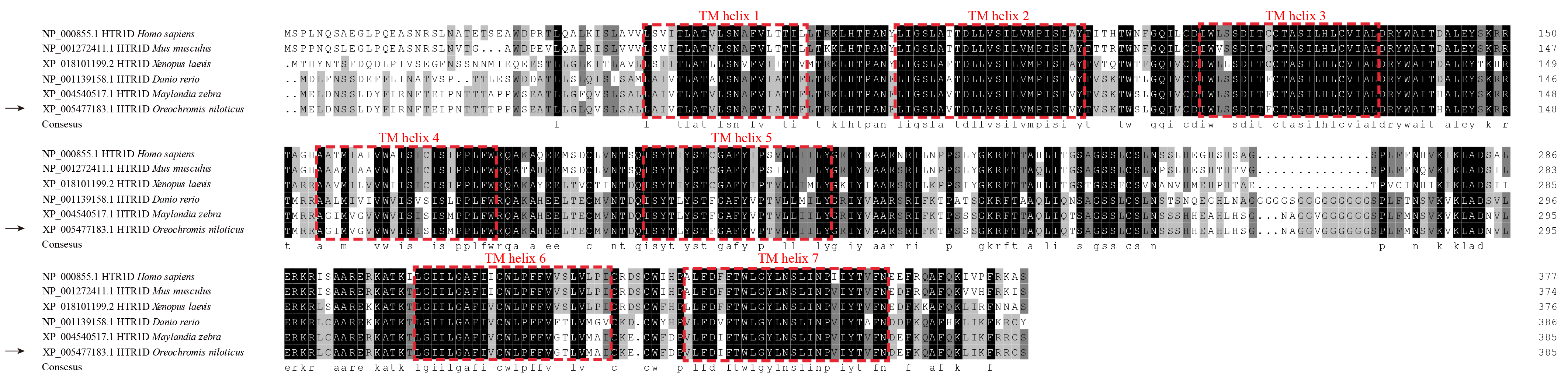

Supplement: Supplementary file 1 [file DataSheet_1.zip › Supplementary materials/Data S1. Multiple sequence alignment/Figure 7-Multiple sequence alignment of HTR1D.tif]

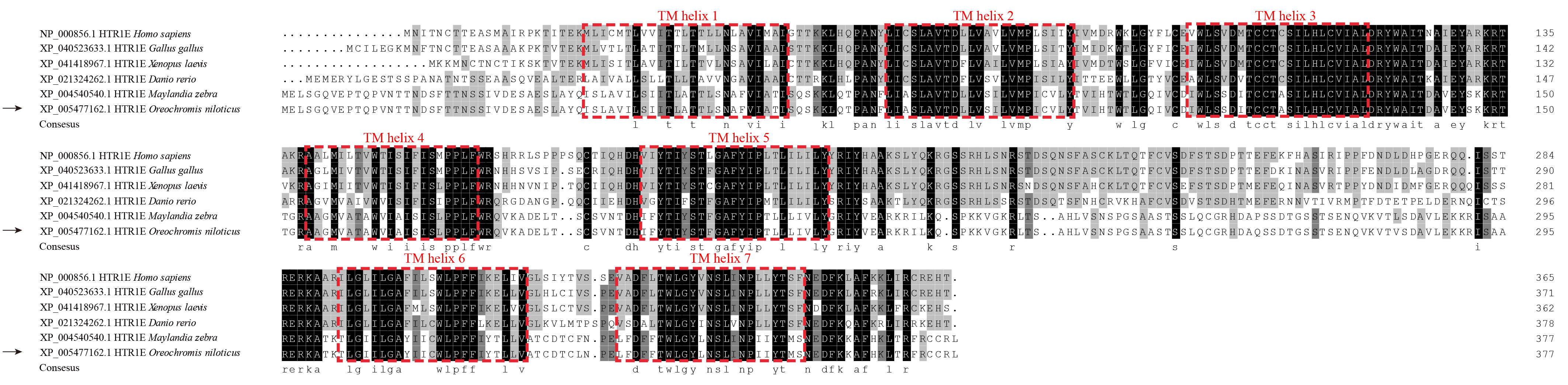

Supplement: Supplementary file 1 [file DataSheet_1.zip › Supplementary materials/Data S1. Multiple sequence alignment/Figure 8-Multiple sequence alignment of HTR1E.tif]

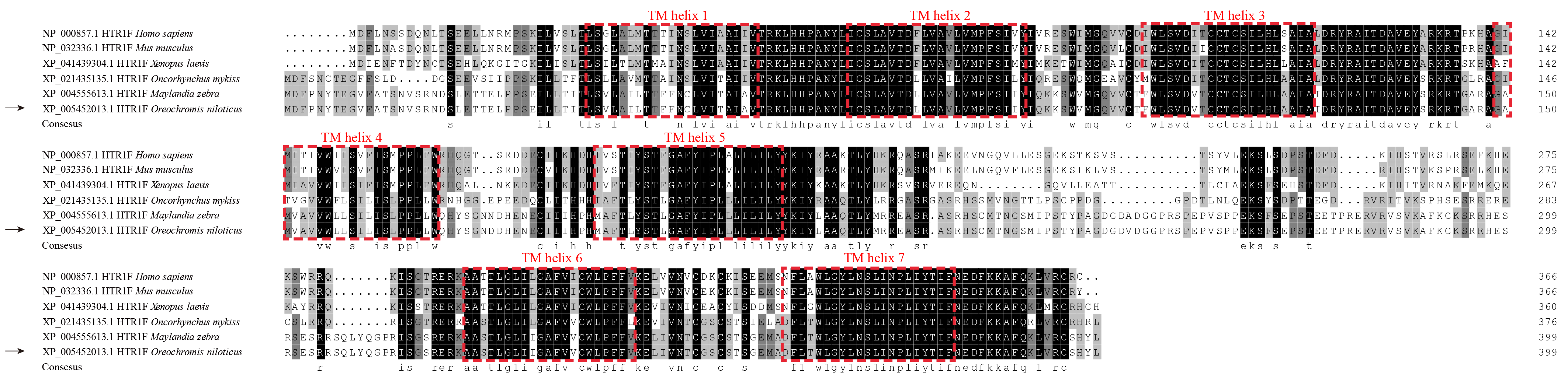

Supplement: Supplementary file 1 [file DataSheet_1.zip › Supplementary materials/Data S1. Multiple sequence alignment/Figure 9-Multiple sequence alignment of HTR1F.tif]

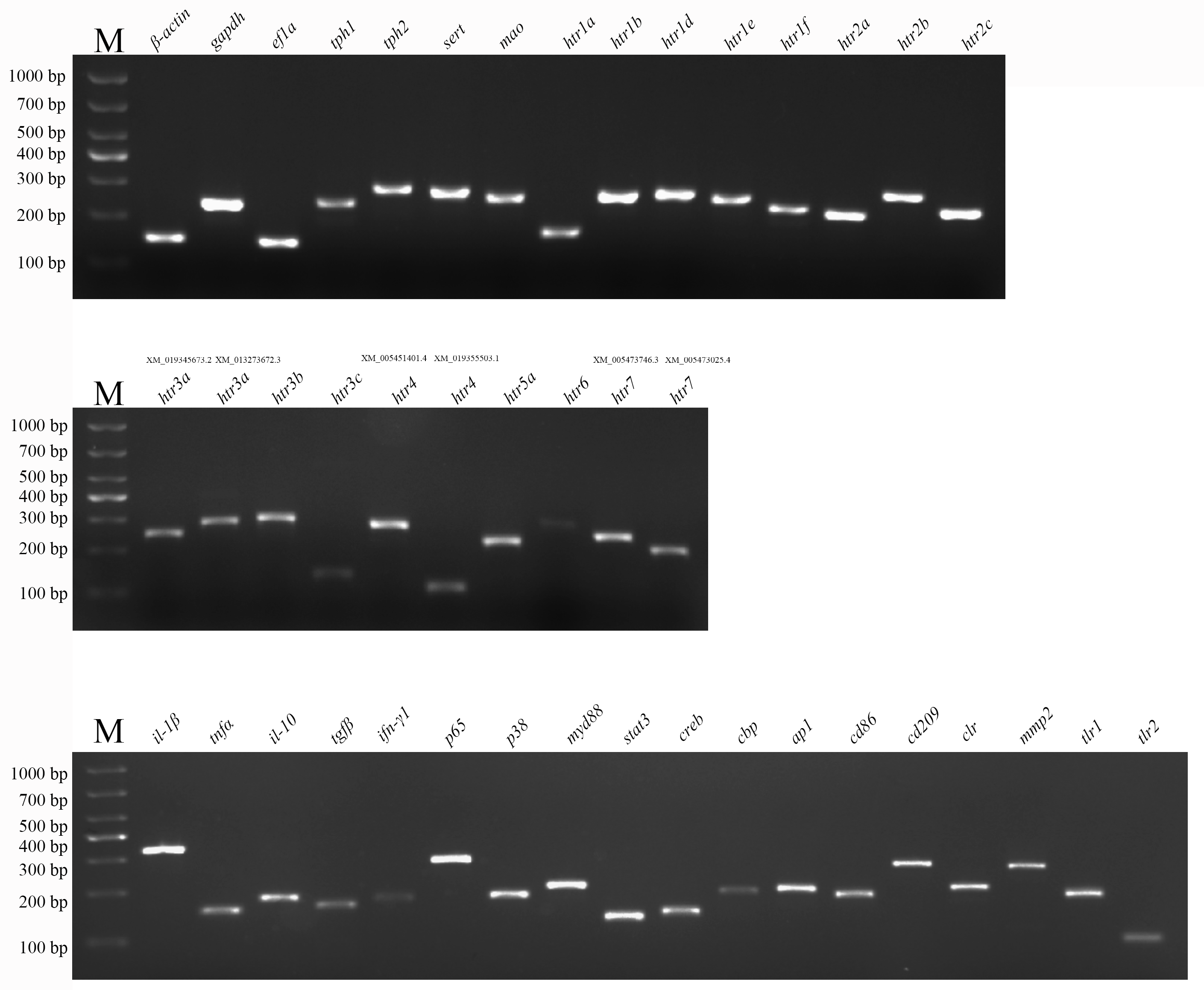

Supplement: Supplementary file 1 [file DataSheet_1.zip › Supplementary materials/Figure S1. Gel electrophoresis of qPCR primers .tif]

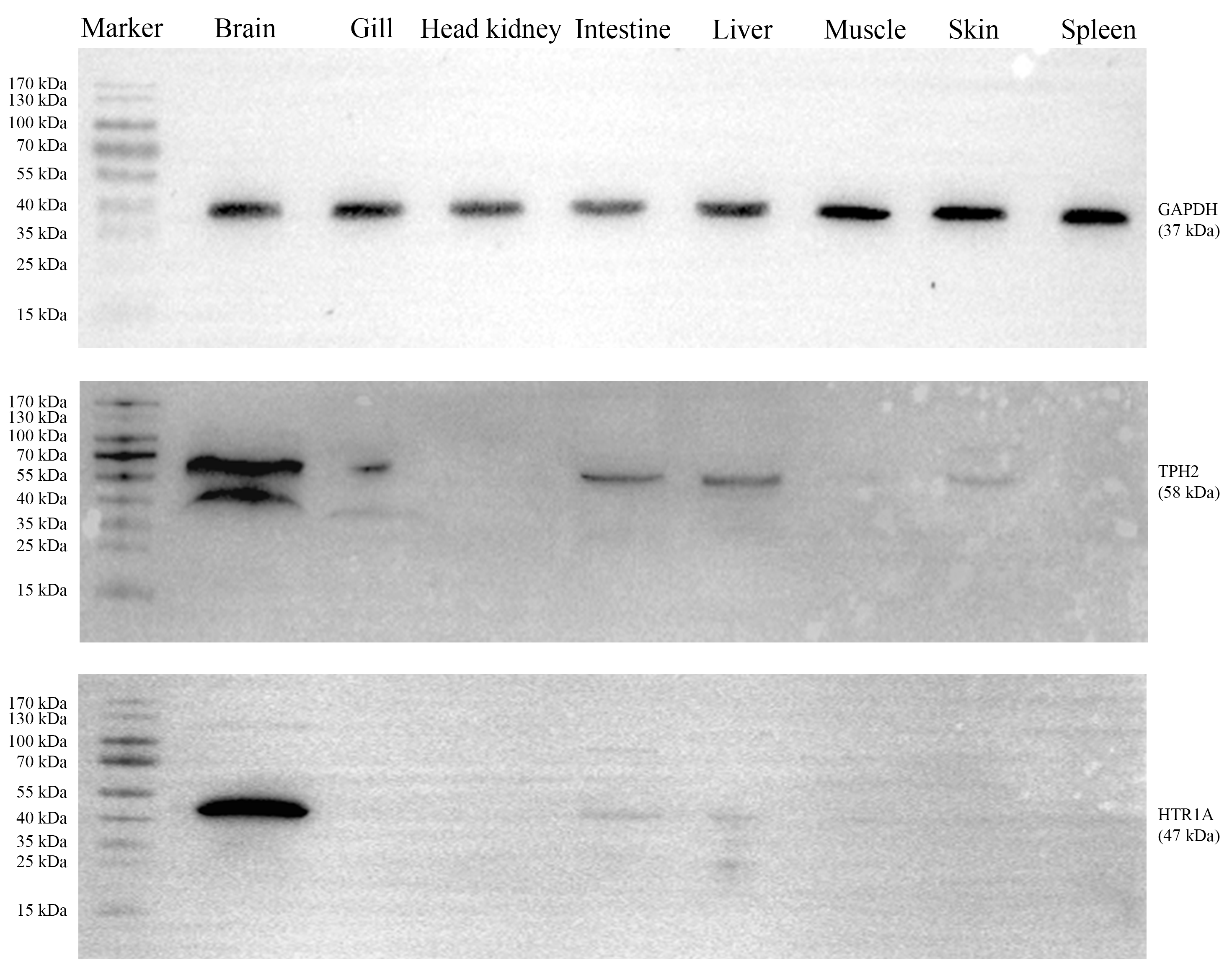

Supplement: Supplementary file 1 [file DataSheet_1.zip › Supplementary materials/Figure S2. Complete figure of Westblot.tif]

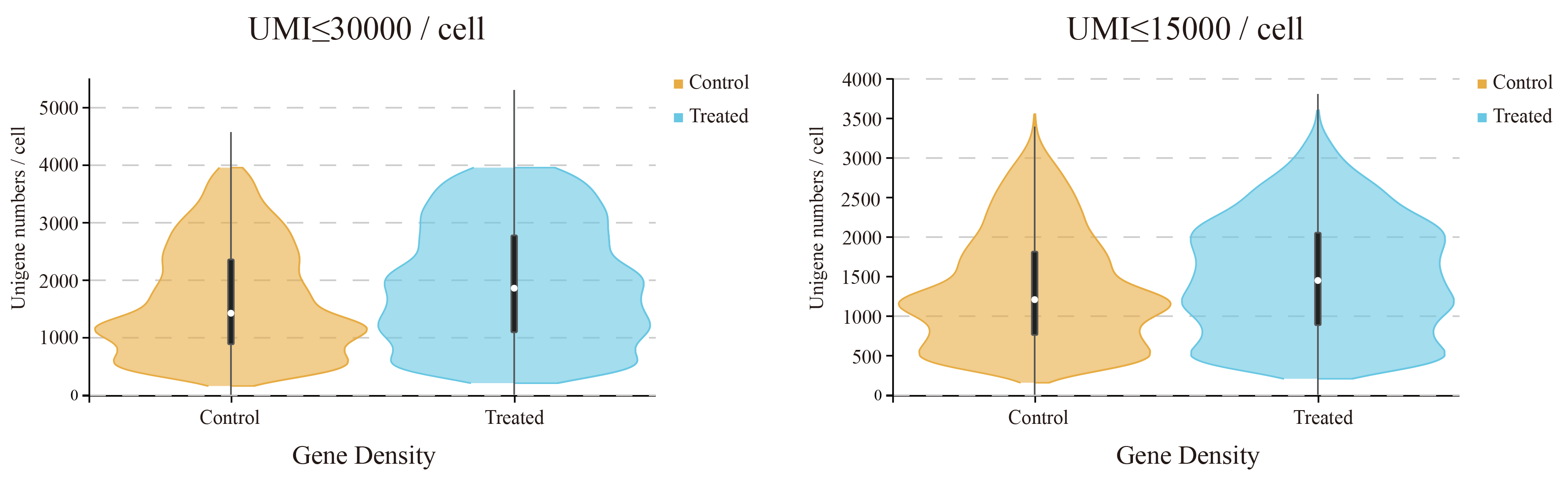

Supplement: Supplementary file 1 [file DataSheet_1.zip › Supplementary materials/Figure S3. Gene density.tif]

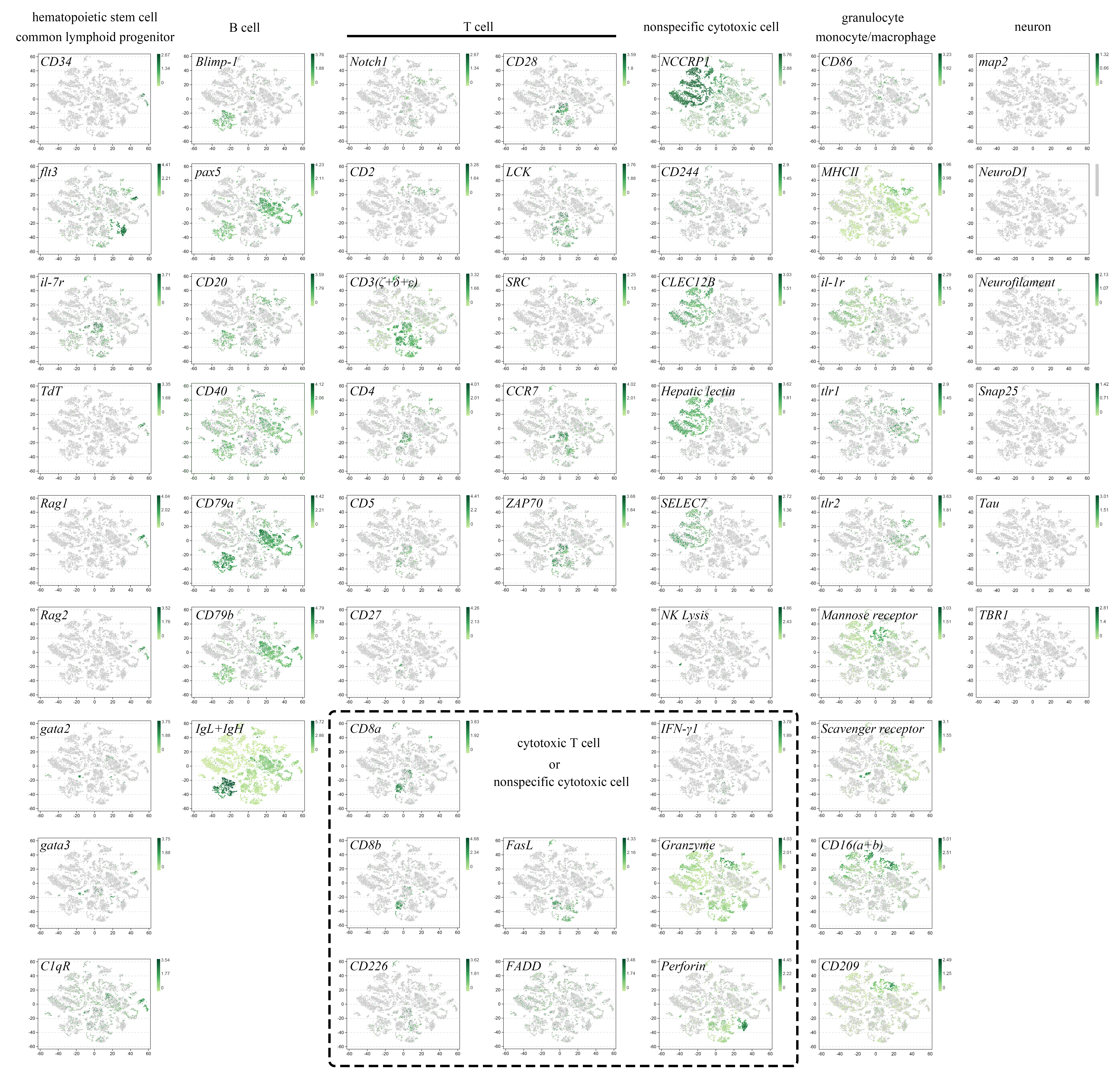

Supplement: Supplementary file 1 [file DataSheet_1.zip › Supplementary materials/Figure S4. Marker genes & Subgroups.tif]

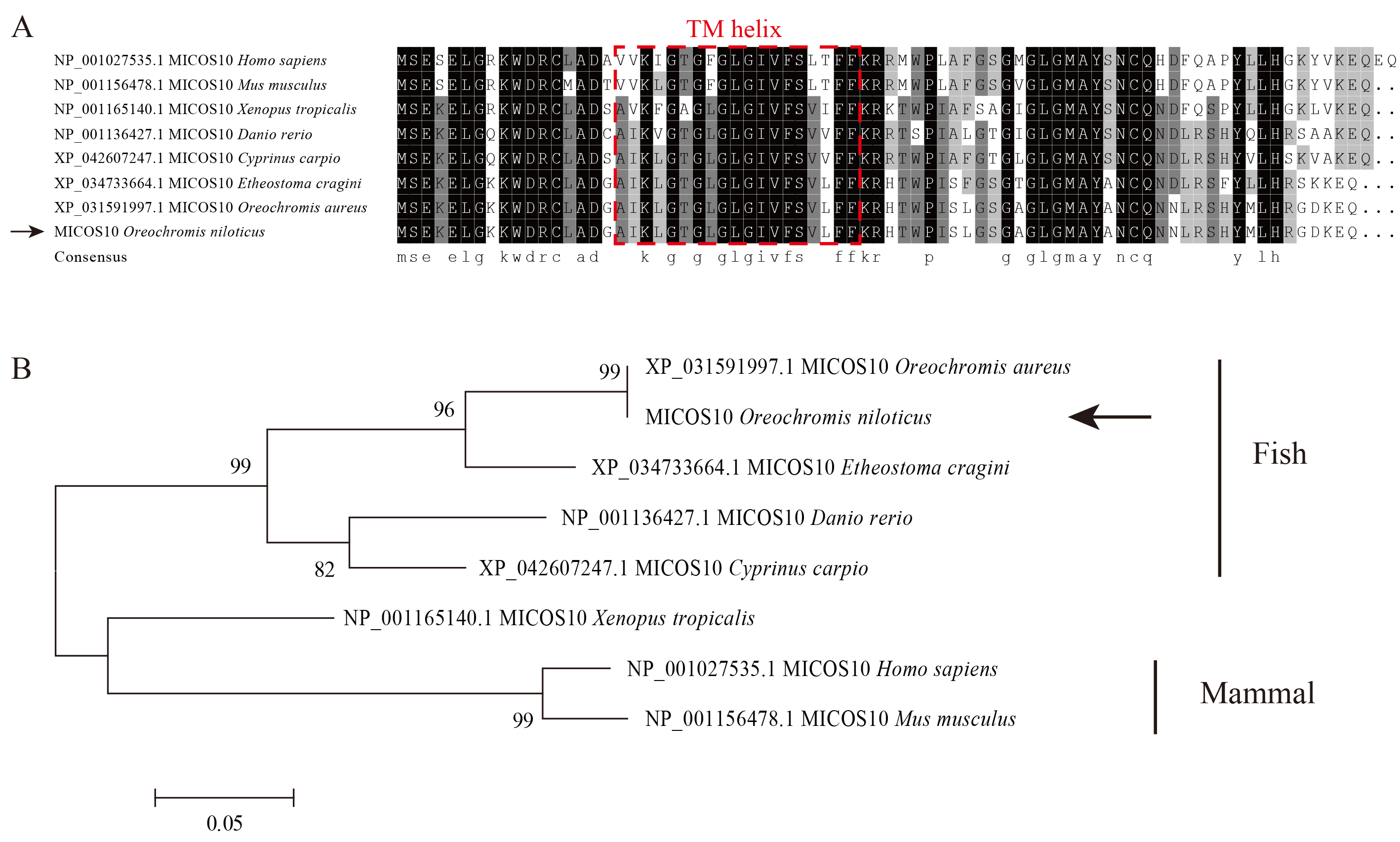

Supplement: Supplementary file 1 [file DataSheet_1.zip › Supplementary materials/Figure S5. Multiple sequence alignment & neighbour-joining (NJ) phylogenetic tree of MICSO10.tif]

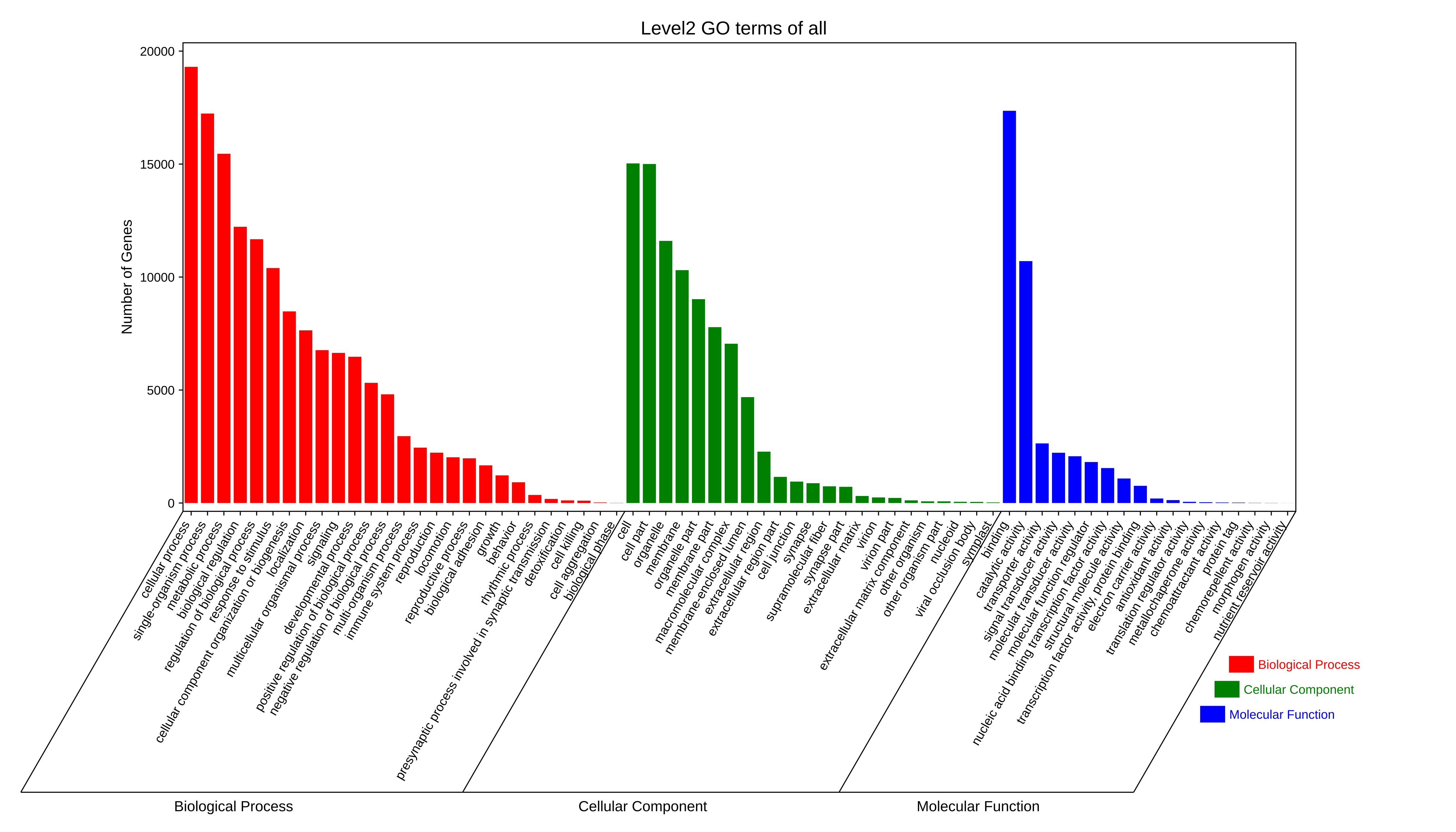

Supplement: Supplementary file 1 [file DataSheet_1.zip › Supplementary materials/Figure S6. GO of HKL (RNA-Seq).png]

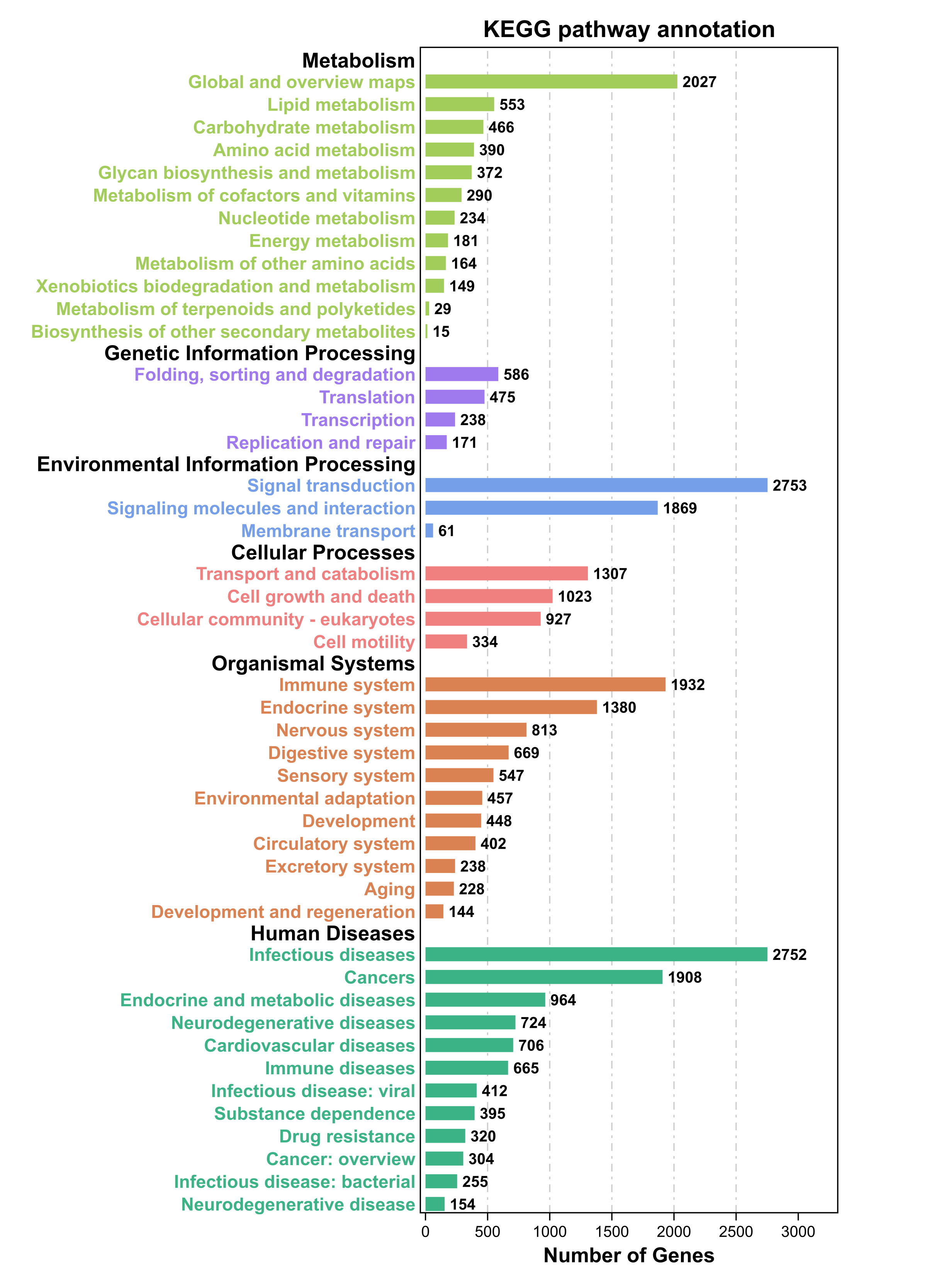

Supplement: Supplementary file 1 [file DataSheet_1.zip › Supplementary materials/Figure S7. KEGG of HKL (RNA-Seq).png]
